# A Structure-Guided Kinase–Transcription Factor Interactome Atlas Reveals Docking Landscapes of the Kinome

**DOI:** 10.1101/2025.10.10.681672

**Published:** 2025-10-14

**Authors:** Ah-Ram Kim, Kerui Huang, Jared L. Johnson, Tomer M. Yaron-Barir, Keven Wang, Lewis C. Cantley, Yanhui Hu, Norbert Perrimon

**Affiliations:** 1 Department of Genetics, Blavatnik Institute, Harvard Medical School, Boston, MA 02115, USA; 2 Department of Cell Biology, Harvard Medical School, Boston, MA 02115, USA; 3 Dana-Farber Cancer Institute, Harvard Medical School, Boston, MA 02115, USA; 4 Department of Pediatrics, Boston Children’s Hospital, Harvard Medical School, Boston 02115, MA 02115, USA; 5 Howard Hughes Medical Institute, Harvard Medical School, Boston, MA 02115, USA

## Abstract

Protein kinases orchestrate cellular processes through phosphorylation, yet the structural basis for their specific binding partner interactions remains largely unmapped. Here, we present a structure-guided atlas of the human and *Drosophila* kinome, built by applying a new interface-aware scoring framework (iLIS) to AlphaFold-Multimer predictions. The resulting atlas recapitulates hallmark sequence preferences, confirms previously reported and functionally related protein-protein interactions (PPIs), and uncovers previously unrecognized docking interactions. Notably, our analysis predicts a potentially widespread docking motif on homeodomain (HD) transcription factors for interaction with basophilic kinases. We validate selected predictions in cells and in vivo and demonstrate the atlas utility by mechanistically dissecting a key pathway controlling lipid metabolism. This resource provides a blueprint for dissecting signaling networks and for the rational design of docking-site-specific kinase modulators.

## Introduction

The precise regulation of cellular processes hinges on protein kinases, which selectively phosphorylate their substrates to control signaling networks. While the importance of the amino acid sequence at the phosphorylation site is well-established^1–5^, a critical layer of specificity is often conferred by transient, structurally defined docking interactions at sites distant from the kinase’s catalytic site^6–8^. These interactions are challenging to capture with conventional proteomic and motif-based methods, leaving a significant gap in our ability to map signaling pathways, predict binding partner selection, and understand how kinase fidelity is precisely achieved.

Advances in deep-learning methods for protein structure prediction, such as AlphaFold^9–11^ and RosettaFold^12,13^, hold the potential to map these interactions at an unprecedented scale^14–22^. However, global confidence metrics for predicted structures are biased toward large, stable interfaces and often miss the subtle, transient docking events that are the hallmark of kinase signaling, particularly those involving intrinsically disordered regions (IDRs)^23,24^. This necessitates an interpretive framework to bridge the gap between predicted structures and functional specificity.

To address this, we developed the integrated Local Interaction Score (iLIS), which identifies high-confidence interactions by combining confidence scores from both the broader interaction interface and the core physical contacts within it. To validate our framework against a challenging and biologically significant system, we applied it at a large scale to the human Ser/Thr (S/T) kinase–transcription factor (TF) interactome, modeling over 240,000 pairs. We chose to focus on kinase interactions with TFs, as they represent a critical nexus between signaling and gene expression^25,26^ and are a protein class rich in the intrinsically disordered regions (IDRs) that mediate transient interactions^23,27,28^, making them an ideal system to challenge our structure-first approach. Analysis of the resulting atlas confirmed the power of our framework, as the predicted structures successfully recapitulated established principles of kinase-partner specificity, with derived in silico interaction motifs aligning well with experimentally defined sequence preferences.

Next, we applied this framework to the full *Drosophila melanogaster* kinome, enabling a binding-defined kinase tree generation. This atlas reveals a wide array of previously unmapped docking sites. A highlight of this analysis is the discovery of a conserved docking motif on homeodomain TFs, which reveals an unanticipated axis of structural regulation. By providing a large-scale structural view that moves beyond simple sequence motifs, this work provides a foundational resource for understanding cellular signaling and lays the groundwork for the rational design of docking site-specific kinase modulators.

## Results

### iLIS enables accurate identification of interactions involving flexible interfaces

**Figure 1.**
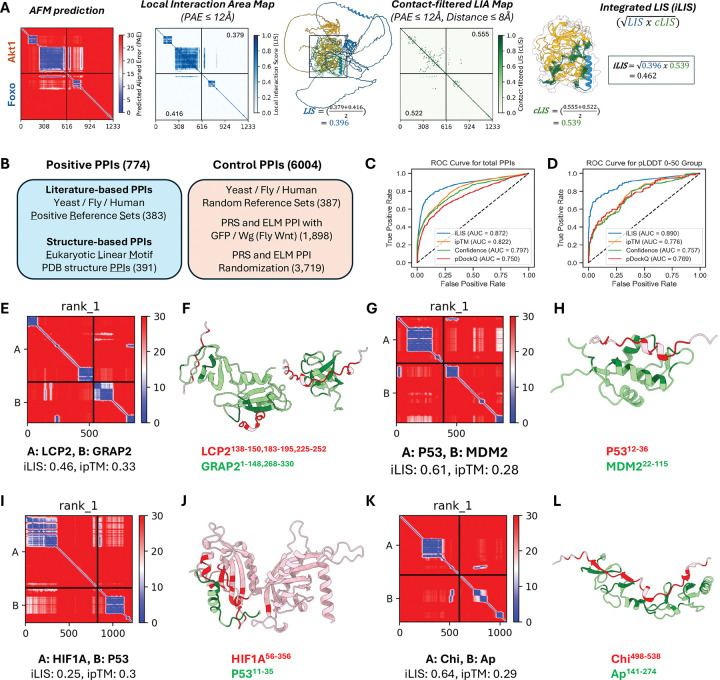
iLIS enables accurate detection of diverse protein-protein Interactions, including those involving flexible interfaces. (A) Schematic illustrating the calculation of the integrated Local Interaction Score (iLIS). AlphaFold-Multimer (AFM) predictions are first analyzed to identify the Local Interaction Area (LIA) within the complex interface, defined by a Predicted Aligned Error (PAE) cutoff (≤12 Å). The LIA is visually represented in the LIA map, where confident interaction domains in the predicted structure are highlighted by a black box. The Local Interaction Score (LIS) is then derived from the LIA by normalizing the PAE values (from the cutoff) into a confidence score between 0 and 1. LIS assesses overall interface confidence by averaging both interfaces. A contact-filtered LIA is established using both the PAE cutoff and a strict physical proximity filter (Cβ–Cβ distance ≤ 8 Å), and confident contacts are visualized by green lines in the structure. The contact-filtered LIS (cLIS) is derived by averaging the individual cLIS scores within this filtered area, focusing on direct physical contact. The geometric mean of LIS and cLIS is finally calculated to yield the robust integrated Local Interaction Score (iLIS), a metric capable of identifying both rigid and transient interactions. *Drosophila* Akt1 and Foxo interaction was used as an example here. (B) Overview of the benchmark dataset, comprising 774 positive PPIs (literature-curated and structure-based) and 6,004 control PPIs (randomized or putatively non-interacting pairs). (C, D) Receiver Operating Characteristic (ROC) curve analysis comparing the performance of iLIS to other global confidence metrics on the full benchmark dataset (C) and the disordered complexes (pLDDT 0–50) (D). (E–L) Representative examples of high iLIS-scoring interactions involving intrinsically disordered regions (IDRs) that are often missed by global metrics. Each pair includes a PAE plot (left) and a structural model (right) highlighting contacting residues. Examples shown are: (E, F) LCP2–GRAP2; (G, H) p53–MDM2; (I, J) HIF1A–p53; and (K, L) Chip–Apterous.

One of the main challenges in using AlphaFold-Multimer (AFM) to predict protein-protein interactions lies in the limitations of global structural confidence metrics, such as the interface-predicted TM-score (ipTM)^10^, Model Confidence^10^ and pDockQ^29^. These metrics often fail to capture interactions mediated by flexible regions like short linear motifs (SLiMs) or intrinsically disordered regions (IDRs)^30^. Although AFM can model such transient complexes with high local accuracy, the presence of extensive non-interacting flexible segments can reduce overall confidence scores, pushing them below commonly accepted thresholds. To overcome this issue, we developed iLIS, a computational framework that builds upon and supersedes our previous work^31^. The iLIS score combines two measures: the Local Interaction Score (LIS; interface confidence within a PAE-defined window of ≤12 Å)^31^ and the contact-filtered LIS (cLIS; confidence restricted to residue pairs meeting both a PAE cutoff (≤12 Å) and a direct Cβ–Cβ contact ≤8 Å) (Fig. 1A; see Sections 1–3 in Supplementary Text).

To evaluate the performance of iLIS, we conducted benchmarking using a diverse dataset of 774 positive and 6,004 control protein-protein interactions (PPIs). This dataset was obtained from literature-curated collections used in evaluating large-scale yeast two-hybrid (Y2H) screens of the yeast, fly, and human proteomes^32–34^, as well as structure-based interactions from Eukaryotic Linear Motif (ELM) database, a resource specializing in the SLiMs that drive many transient PPIs^35^ (Fig. 1B; see Supplementary Text, Sections 4–5, for dataset details). On this benchmark, iLIS significantly outperformed existing global confidence metrics like ipTM, Model Confidence, and pDockQ, achieving a ROC AUC of 0.872 versus 0.822, 0.797, and 0.750, respectively (Fig. 1C).

We then compared iLIS to two other recent local confidence metrics, actifpTM^36^ and ipSAE^37^. While all three local metrics are highly correlated and demonstrate similarly strong performance in distinguishing positive from control interactions (Supplementary Fig. 1A–F), we chose to proceed with iLIS because it holds a modest but consistent advantage in its ability to statistically distinguish between positive and control datasets (Supplementary Fig. 1G, H) and to recover more true positives at a 10% false positive rate (Supplementary Fig. 1I, J). This improved sensitivity is most apparent in challenging cases, such as highly disordered complexes (pLDDT 0–50), where local metrics are essential. iLIS maintained a high predictive accuracy (AUC = 0.890), whereas the performance of global metrics such as ipTM dropped sharply (AUC = 0.776) (Fig. 1D). Furthermore, iLIS demonstrated robust generalizability, outperforming ipTM on a time-split benchmark of structures deposited after AFM’s training date (Supplementary Fig. 1K–M). Taken together, these results highlight iLIS as a reliable and well-suited metric for identifying the transient, flexible interactions central to our study.

To visualize this feature, we highlight several bona fide interactions from our benchmark datasets that are scored highly by iLIS but poorly by ipTM (Supplementary Fig. 2). We defined our high-confidence thresholds at a 10% False Discovery Rate (FDR) using literature-derived Y2H reference sets, yielding cutoffs of iLIS ≥ 0.223 and ipTM ≥ 0.48. The PAE plots for these and additional examples all share a characteristic signature of localized confident interaction within complexes with uncertain global structures. Four such representative cases are visualized in Figure 1E to 1L. The AFM model of the LCP2-GRAP2 complex (iLIS = 0.46, ipTM = 0.33)—an interaction with a known crystal structure^38^—is correctly identified by iLIS despite a low ipTM score caused by extensive flexible regions. Similarly, iLIS successfully identifies the critical interface of the canonical p53-MDM2 interaction (iLIS = 0.61, ipTM = 0.28)^39^, where the small, structured binding motif on p53 is obscured by large disordered domains. This capability extends beyond structurally characterized pairs to known physical interactions, such as HIF1A and p53 (iLIS = 0.25, ipTM = 0.3)^40,41^, and previously mapped *Drosophila* transcription factor interactions like Chip and Apterous (iLIS = 0.64, ipTM = 0.29)^42–44^. These examples underscore the strength of iLIS in rescuing functionally critical interactions mediated by localized interfaces, a class of interactions essential to cell signaling that global metrics systematically overlook.

### Architecture and validation of the human kinase-transcription factor interactome

Having validated iLIS to identify transient, motif-driven interactions, we applied it to construct a comprehensive structural atlas of the human Serine/Threonine (S/T) kinase–transcription factor (TF) interactome. We systematically modeled over 240,000 interactions between 207 S/T kinases and 1,162 TFs. The complete set of predicted interactions, their corresponding iLIS, and the predicted residue-level interfaces for this human kinase-TF screen are provided in **Supplementary Data 1**.

**Figure 2.**
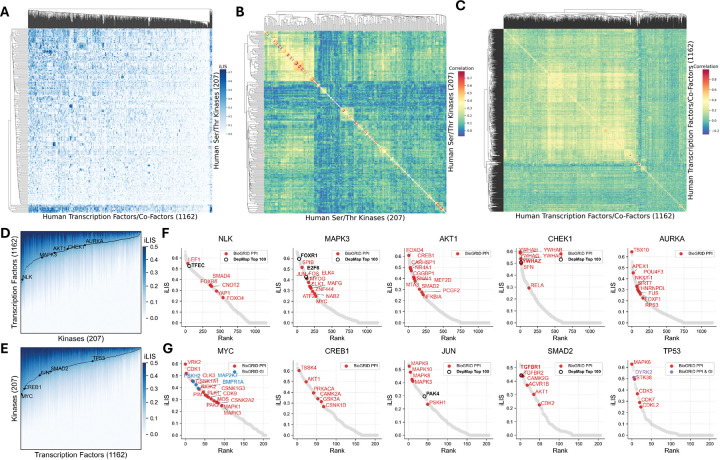
Architecture and validation of the human kinase-transcription factor interactome. (A) A comprehensive interactome of all 240,000+ predicted human S/T kinase-TF interactions. (B) Kinase correlation map by transcription factor interaction profile. (C) TF correlation map by kinase interaction profile. (D) Accumulated iLIS distribution plot of human kinases against transcription factors. Highlighted hub kinases are indicated (NLK, MAPK3, AKT1, CHEK1, AURKA). (E) Accumulated iLIS distribution plot of human TFs against kinases. Highlighted transcription factors are indicated (MYC, CREB1, JUN, SMAD2, TP53). (F) Example AFM screens of human S/T kinases against human transcription factors/co-factors. For the screens in both (F) and (G), previously reported BioGRID interactions are indicated as colored dots: Protein-Protein Interactions (PPI, red), Genetic Interactions (GI, blue), or both (purple). A black ring highlights partners that rank among the top 100 functionally co-dependent genes (ranked by absolute correlation) for that specific kinase/TF in the DepMap CRISPR screens. Highlights for external data are shown only for predictions meeting the threshold of iLIS >= 0.223. (G) Example AFM screens of human transcription factors against human S/T kinases.

The resulting interactome reveals a functionally coherent landscape where proteins with similar binding partners cluster together, correctly grouping known kinase and TF families (Fig. 2A; Supplementary Fig. 3A). This structure-based organization is particularly evident in our correlation analysis, which correctly clusters kinases such as the MAPKs and CDKs, and transcription factors such as the HOX family and FOXO family into their respective functional groups (Fig. 2B, C; Supplementary Fig. 3B, C). The network displays a hub-and-spoke architecture, with kinases like NLK and MAPK3 acting as master regulators and TFs like MYC and TP53 serving as key integrators (Fig. 2D, E).

To validate these predicted interactions, we cross-referenced the atlas against two orthogonal datasets. Our predictions recovered many known physical interactions from the BioGRID database^45^ (Fig. 2F, G; Supplementary Fig. 4; colored dots). For functional validation, we integrated our data with the Cancer Dependency Map (DepMap), which maps gene co-dependencies across diverse cancer cell lines from CRISPR screens^46^. We found that a number of our high-confidence structural predictions rank among the top 100 functionally co-dependent partners, as determined by absolute correlation score, in the DepMap dataset (25Q2), providing crucial functional support for these specific pairs and reinforcing the biological significance of the atlas. Beyond validation, the atlas serves as a powerful discovery tool: by integrating our structural predictions with known physical and genetic links, it pinpoints high-priority candidates for uncovering novel mechanisms of kinase-mediated transcriptional regulation.

### Computational motif discovery recapitulates key principles of kinase-partner specificity

**Figure 3.**
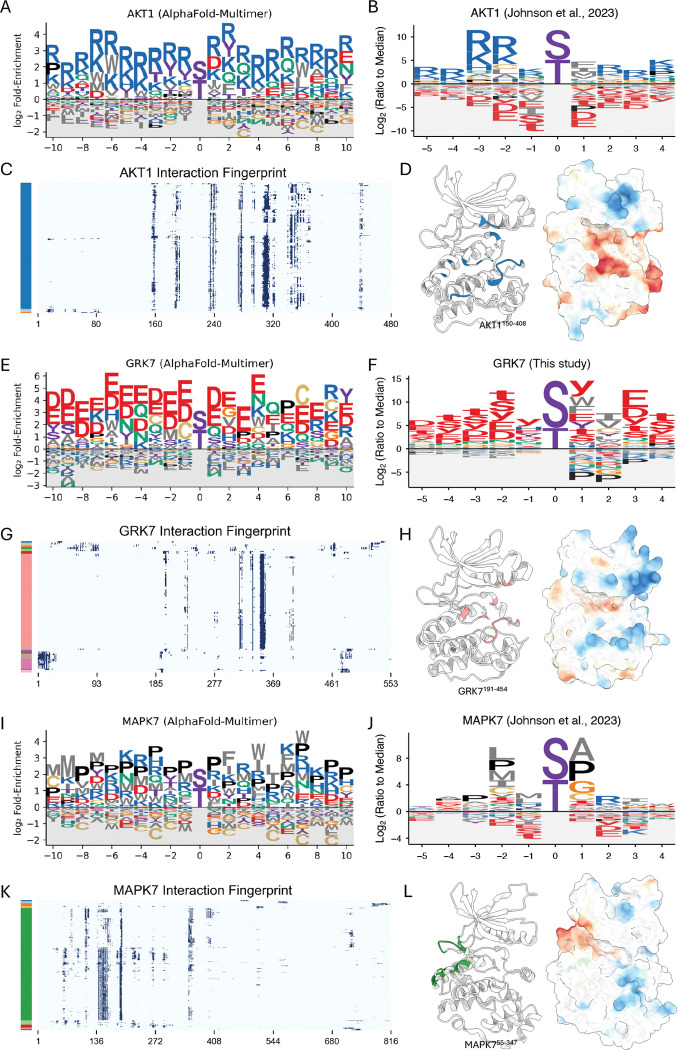
Computational motif discovery recapitulates key principles of kinase-partner specificity. (A, E, I) Computationally predicted Position Weight Matrix (PWM) for human AKT1, GRK7, and MAPK7 derived from AlphaFold-Multimer interfaces. (B, F, J) Experimentally determined PWM for human AKT1 (Johnson et al., 2023), GRK7 (This study), and MAPK7 (Johnson et al., 2023). Lowercase letters (s, t, y) denote the phosphorylated residue. (C, G, K) Interaction fingerprint of AKT1, GRK7, and MAPK7. The y-axis represents individual transcription factor partners, which are clustered based on their binding footprint on the kinase (x-axis). Color bars on the left indicate the resulting partner clusters. (D, H, L) Structural mapping of interaction hotspots. For each kinase, the left structure highlights the primary interaction hotspot, defined as residues that form contacts in at least 50% of the partners from the largest cluster in the fingerprint analysis. The right structure shows the electrostatic potential mapped to the surface (red: acidic; blue: basic). Notably, for MAPK7 (L), these hotspot residues (green) map to the conserved docking groove used for Kinase Interaction Motif (KIM) binding, a site distant from the catalytic cleft.

From the global architecture of the kinase-TF network, we next sought to determine the structural principles that govern its interaction specificity. To do this, we derived interaction motifs from the structural interfaces for three representative kinase families (see Supplementary Text, Section 6 for methodological details). For the basophilic kinase AKT1, our computationally derived Position Weight Matrix (PWM) (Fig. 3A) shows strong agreement with the known experimental motif (Fig. 3B)^4^, successfully recapitulating the hallmark enrichment for basic R and K residues. An interaction fingerprint highlights a specific hotspot region on the kinase (Fig. 3C), which corresponds to the catalytic cleft—enriched with acidic residues poised to engage the basic motifs of its partners—when visualized on the structure (Fig. 3D). This principle of R/K enrichment was broadly conserved across other basophilic kinases, including PKA, AKT2, PIM1, and all CDKL family members (Supplementary Fig. 5A).

This principle of electrostatic complementarity is also evident for the acidophilic kinase GRK7. Our predicted PWM (Fig. 3E) shows a strong enrichment for the acidic residues D and E. This prediction is strongly supported by our new experimental profiling of GRK7, which confirms a clear preference for acidic phosphosites surrounding the phosphorylation site (Fig. 3F; Supplementary Fig. 6). The interaction fingerprint identifies specific hotspot residues (Fig. 3G), and the structure reveals that these hotspots form the catalytic cleft, which is lined with basic residues (Fig. 3H). This acidic preference was uniformly predicted across the entire GRK family and other acidophilic kinases, such as CSNK1 isoforms (CSNK1A1 and CSNK1G1) (Supplementary Fig. 5B).

Finally, for the proline-directed kinase MAPK7, our analysis identifies the characteristic +1 Pro preference, consistent with experimental data^4^, and a broader Pro enrichment in flanking positions (Fig. 3I, J). Notably, the interaction fingerprint and structure analysis for MAPK7 show that the primary hotspots are not located in the catalytic cleft but elsewhere on the kinase surface (Fig. 3K, L). Mapping these sites onto the structure confirms they correspond to the conserved docking groove used for Kinase Interaction Motif (KIM) binding—a mode of partner engagement separate from the active site^47–49^. This +1 Pro feature and broad Pro enrichment were also observed in other MAPK family members (Supplementary Fig. 5C).

While our computationally derived PWMs show general agreement with experimental data, minor deviations are observed. These may reflect the inherent limitations of our modeling, which is purely structural, considers only binary interactions (thereby excluding interactions that depend on cofactors or bridging proteins), and does not account for other cellular factors like post-translational modifications that are not captured in our models. Overall, these findings highlight the utility of a structure-based approach for large-scale motif discovery and suggest that the combination of AFM and our iLIS framework can capture key principles of kinase-partner specificity, providing a foundation for discovering novel interaction modes and regulatory networks. The full dataset containing these computationally derived PWMs for all human S/T kinases is available in **Supplementary Data 2**.

### An interactome-based kinase tree reveals binding-defined subfamilies

To systematically organize these binding principles at a systems level, we next applied our framework to the complete kinome of *Drosophila melanogaster*, performing a systems-level screen where we modeled interactions between 252 kinases (S/T, Tyr, and pseudokinases) and 841 TFs/co-factors. The resulting interactome provides a global view of kinome-TF connectivity (Supplementary Fig. 7), revealing a highly structured landscape where kinases with similar binding profiles form distinct groups. Furthermore, the network displays significant variability in connectivity, with some kinases acting as promiscuous hubs while others are highly selective, a feature also observed in our human kinome analysis. The complete prediction results for this screen, including iLIS and residue-level interfaces, are provided in **Supplementary Data 3**.

**Figure 4.**
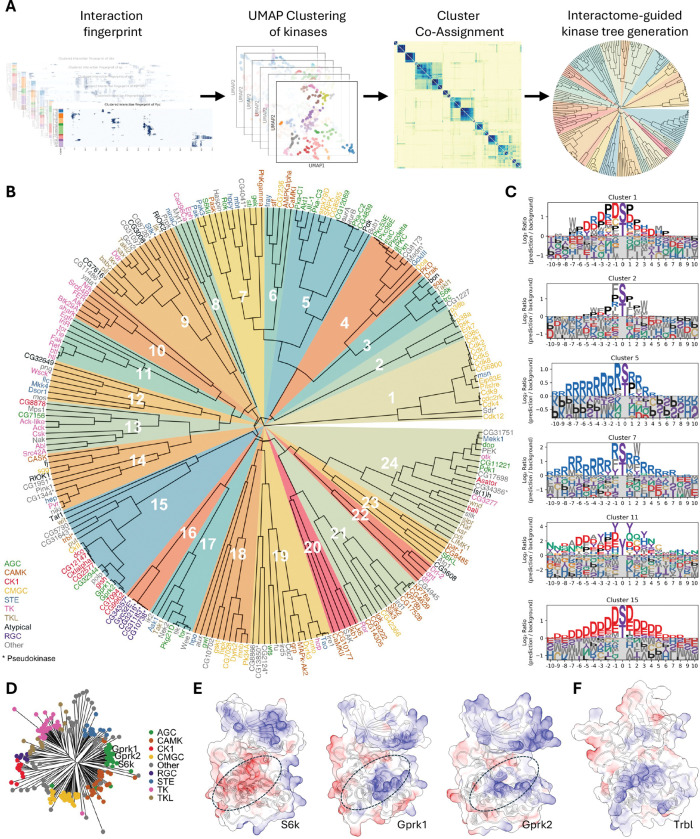
An interactome-based kinase tree reveals binding-defined subfamilies and refines functional classifications. (A) Pipeline for constructing the interactome-guided kinase tree. A “TF-binding fingerprint” for each kinase is subjected to a stability-based clustering analysis (10,800 runs) to generate a consensus matrix, which is then used for hierarchical clustering to build the final tree. (B) Binding-defined kinase tree, organizing 231 *Drosophila* kinases based on their TF-binding fingerprints. Kinases are color-coded by their sequence-based family, with pseudokinases marked by an asterisk (*). Numbered wedges indicate the binding-defined clusters. (C) Representative Position Weight Matrices (PWMs) illustrating the binding preferences for key functional clusters, such as proline-directed (e.g., Cluster 2), basophilic (e.g., Cluster 5), and acidophilic (e.g., Cluster 15) groups. The full atlas of cluster motifs is in Supplementary Figure 8. (D) Sequence homology-based kinase tree, which groups Gprk1 and Gprk2 with the basophilic AGC family. (E) Structural basis for the reclassification of Gprk1 and Gprk2. Although sequence-homologous to basophilic kinases like S6k (acidic cleft, red), their binding fingerprints place them in the acidophilic Cluster 15. This is explained by their basic catalytic clefts (circled, blue), which are suited for acidic partners. (F) The pseudokinase Tribbles (Trbl) clusters with the acidophilic group (Cluster 15). Its electrostatic surface reveals a basic pseudoactive site (circled), suggesting it retains the ability to bind acidic partners.

To move beyond this landscape and define binding principles, we selected the 231 kinases with sufficient data coverage for a robust clustering analysis. For this subset, we developed a pipeline using unsupervised learning (Fig. 4A; see Supplementary Text 7 for details). To classify kinases by their binding preferences, we first generated a quantitative ‘TF-binding fingerprint’ for each kinase. These fingerprints—each one a high-dimensional vector derived from interaction hotspot data (as visualized in Fig. 3C, G, and K)—were then assembled into a large input matrix (231 kinases x 219,796 features) where the features represent individual TF residues.

Because dimensionality reduction with the Uniform Manifold Approximation and Projection (UMAP) algorithm^50^ can produce variable results depending on initial conditions and hyperparameters, we employed a stability-based approach. We performed over 10,800 clustering runs, each initiated by a UMAP projection with varying parameters and bootstrapped features, and aggregated the results into a final consensus matrix. This consensus matrix robustly quantifies kinase similarity. We then used this matrix to build the final binding-defined kinase tree via hierarchical clustering. The resulting tree organizes the kinome by binding preference rather than by the sequence homology of kinase domains, helping to resolve inconsistencies in sequence-based classifications and suggesting latent functions of non-catalytic kinases (Fig. 4B).

This classification provides a detailed look at the kinome’s organization (Fig. 4C). The proline-directed kinases, for example, largely composed of the CMGC family, separate into two distinct superclusters (Clusters 1 and 2). The CDK family (e.g., Cdk1, Cdk2) forms one group that, in addition to a core proline preference, is distinguished by an enrichment for charged residues (Cluster 1). In contrast, the MAPK family (e.g., Rl, p38a) forms a second group with a clear preference for proline at canonical positions but less overall charge, instead featuring hydrophobic W and F residues (Cluster 2). Our analysis also identified a larger neighborhood of basophilic kinases, which separated into several distinct groups (Clusters 3–7). For instance, Cluster 5 (including Pka-C1, Akt1) shows a preference for a Proline at the +1 position, while Cluster 7 (a mixed group of AGC, CAMK, and STE kinases) lacks this and instead prefers hydrophobic W and F residues. Finally, tyrosine kinases formed two neighboring clusters (Clusters 10 and 11), with receptor tyrosine kinases in Cluster 11 showing a preference for tyrosine and acidic residues. Thus, the binding-defined tree not only sorts the kinome into major superfamilies but also reveals subtle, biochemically distinct binding preferences that subdivide these large classes.

### The interactome tree refines sequence-based kinase classifications

Sequence-based trees chart evolutionary history but can group kinases with divergent binding preferences and leave some families ambiguous (Fig. 4D). Clustering by interaction fingerprints offers a complementary, biochemical view, often grouping kinases from distinct sequence clades into binding-defined clusters (e.g., the mixed AGC/CAMK/STE group in Cluster 7). Our tree thus provides a framework for re-evaluating kinase family definitions and assigning previously unclassified kinases.

A compelling example of this re-evaluation is the G-protein-coupled receptor kinase (GRK) family. While sequence-based annotations place the fly GRKs, Gprk1 and Gprk2, within the predominantly basophilic AGC family (Fig. 4D)^51^, our interaction fingerprint analysis clusters them with canonical acidophilic kinases like the CK1 family and Polo (Cluster 15). Consistent across species, our analysis of human GRKs also revealed a clear acidophilic motif (Fig. 3E, Supplementary Fig. 5B) that is in strong agreement with large-scale experimental motif data^4^ and, crucially, with our new experimental data for GRK7 presented in this study (Fig. 3F, Supplementary Fig. 6). This finding aligns with previous functional studies showing that GRKs are, in fact, functionally acidophilic^52,53^. Our structural models of Gprk1 and Gprk2 provide a mechanistic basis for this finding, revealing a catalytic cleft lined with basic residues poised to bind acidic substrates (Fig. 4E).

Furthermore, because our approach is based on structurally predicted binding, it provides a novel means to characterize pseudokinases, whose binding preferences around the pseudoactive site cannot be identified by traditional phosphotransfer assays. For instance, the well-known pseudokinase Tribbles (Trbl), a member of the CAMK family, clusters with the acidophilic kinase group (Cluster 15). This suggests that Trbl retains specific binding preferences for acidophilic partners by utilizing a basic surface on its pseudoactive site (Fig. 4F), allowing it to function as a ‘pseudosubstrate-like decoy’ or competitive inhibitor^54–56^. By binding the same targets as active acidophilic kinases, Trbl may act as a kinase antagonist, shaping pathway output.

Finally, our tree provides a framework for classifying kinases with ambiguous assignments. An example is the Aurora kinase family (AurA/AurB). While traditional sequence homology places them in an ‘Other’ group^57,51^ and a structure-based multiple sequence alignment reassigns them to the CAMK family^58^, our binding-defined tree offers a third, distinct classification. In our analysis, Aurora kinases sort into the basophilic Cluster 5, alongside kinases from the AGC and CMGC families.

In summary, this interactome-based approach provides a robust framework for classifying kinases by their binding profiles. It offers a functional view that complements traditional sequence-based trees by largely recapitulating the major kinase families while also revealing subtle subgroup preferences, as exemplified by the reclassification of the GRKs. These results suggest that this interaction fingerprinting strategy could be a broadly applicable tool for classifying other large protein families.

### A predicted docking motif suggests a conserved link between basophilic kinases and homeodomain transcription factors

**Figure 5.**
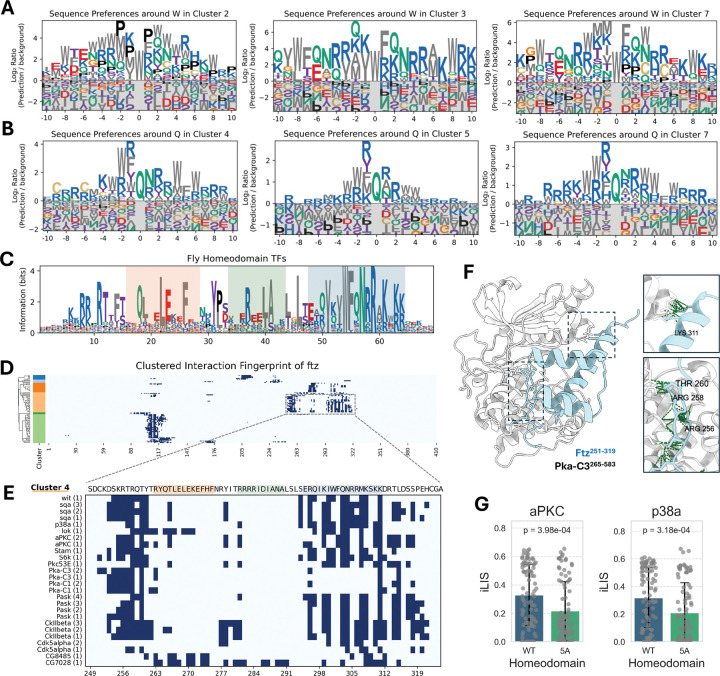
A Conserved docking motif provides a structural link between homeodomain transcription factors and kinases. (A, B) Identification of non-catalytic docking features. A survey of PWMs from multiple kinase clusters revealed recurring enrichment for flanking W, F, Q, and N residues. Representative motifs centered on W (A) and Q (B) are shown. (C) Sequence logo of a conserved composite motif identified in Homeodomain (HD) transcription factors. The logo, derived from all *Drosophila* HD proteins, highlights the consensus for the domain’s three α-helices (helix 1: pale orange; helix 2: pale green; helix 3: pale blue). (D) Clustered interaction fingerprint for the HD protein Fushi tarazu (Ftz). Kinases (y-axis) are clustered by their interaction patterns, revealing the homeodomain as a major hub. The boxed region is shown in detail in (E). (E) Focused view of the Ftz homeodomain fingerprint revealing two distinct interaction hotspots: a primary site near the N-terminus and a second corresponding to the composite motif on the third α-helix, where Lysine 311 (K311) acts as a key anchor point. Kinases are listed with their corresponding model rank. (F) Structural model of the Pka-C3–Ftz complex illustrating a two-pronged binding mechanism. The kinase’s catalytic cleft engages the N-terminal region (bottom inset), while a separate surface contacts the composite motif on the third α-helix (top inset). (G) *In silico* mutagenesis of five key basic residues to Alanine (5A) in the auxiliary motif was predicted to significantly reduce interaction strength (iLIS) with both the basophilic kinase aPKC and the MAPK p38a.

While analyzing the canonical motifs from our kinase tree, we noticed that several clusters displayed unexpected preferences for residues in their flanking regions. For instance, the proline-directed Cluster 2 and the basophilic Cluster 7 both showed a distinct enrichment for hydrophobic F (phenylalanine) and W (tryptophan) residues (Fig. 4C). This prompted a systematic survey of our complete set of PWMs, which confirmed that motifs from multiple basophilic and MAPK clusters had recurring spikes for W, F, and Q/N in these regions (Fig. 5A, B; Supplementary Fig. 8; **Supplementary Data 4**). Observing that these modest signals often appeared linked, we hypothesized they might form a composite docking motif.

To test this hypothesis, we performed a proteome-wide scan for this constellation of residues. The search identified a highly specific composite motif, [V/I]xxWFQNRRx[K/R]x[K/R][K/R], which maps precisely to the third α-helix of homeodomain (HD) transcription factors. A consensus sequence derived from all *Drosophila* HD TFs confirms the strong conservation of this core motif (Fig. 5C).

Filtering our atlas for HD-containing proteins revealed a broad regulatory network. Numerous kinases from multiple families form high-confidence interactions with dozens of HD TFs, with basophilic kinases and MAPKs being particularly enriched among the top interactors in flies (Supplementary Fig. 9A–C). This kinase-HD network appears to be deeply conserved, as a similar analysis of our human interactome revealed a widespread and enriched set of interactions (Supplementary Fig. 9D–E).

To explore the functional implications of this network, we focused on Fushi tarazu (Ftz), a key homeodomain transcription factor required for embryonic segmentation in *Drosophila*^59–62^. The Ftz protein is known to be dynamically phosphorylated *in vivo* at numerous sites during different stages of development^63,64^. Our interaction fingerprint confirms that its homeodomain is a major interaction hub for numerous kinases (Fig. 5D). A detailed view of this region reveals two primary, spatially distinct interaction hotspots (Fig. 5E): the first is near the N-terminus of the homeodomain around a known PKA phosphorylation site^64^, while the second corresponds to the newly identified composite motif on the third α-helix.

Our structural model of the Pka-C3–Ftz complex provides a mechanistic basis for this dual-site interaction (Fig. 5F). The model shows the kinase’s catalytic cleft engaging the N-terminal phosphorylation region, while a separate surface on the kinase contacts the C-terminal composite motif on the third α-helix. This suggests a model where the N-terminal region serves as the primary catalytic site, while the conserved basic patch within the newly identified composite motif is predicted to act as an auxiliary docking site to enhance binding affinity and specificity. This two-pronged binding mode appears to be a general feature, as similar dual interaction hotspots were observed for numerous other HD TFs in both fly and human (Supplementary Fig. 10).

To test the functional importance of the conserved basic patch within the C-terminal composite motif, we performed *in silico* mutagenesis of its five key basic residues to Alanine (5A). We screened these 5A mutants from all fly HD proteins against aPKC and p38a, the top-enriched HD-interacting kinases. The models predicted that the 5A mutation would broadly disrupt these interactions, as reflected by a significant reduction in iLIS scores (Fig. 5G). This result supports the important role of the auxiliary basic patch in mediating a stable interaction, implying that engagement of the N-terminal phosphorylation site alone is insufficient to do so.

This finding reveals a potential mechanism for integrating signaling inputs with transcriptional control. While the third α-helix is essential for DNA binding^65–69^, it is also a known interface for PPIs with other cofactors^70–72^. Our data now add a critical new dimension to its function: our model suggests that the conserved basic patch within this composite motif serves as a critical auxiliary docking interface for kinases, positioning them for the phosphorylation of residues on the N-terminal region adjacent to HD. This suggests a model where kinases dock onto a surface that is bifunctional—mediating both DNA binding and kinase recruitment—positioning them to phosphorylate their targets and regulate the transcriptional activity of the HD protein. Mutations in this critical region are therefore likely to have complex effects by disrupting both auxiliary kinase docking and DNA recognition, which may explain the high prevalence of pathogenic variants at this site^69,73^.

### The kinome atlas systematically identifies inhibitable allosteric hotspots

A central challenge in drug development is designing selective kinase inhibitors, as the ATP-binding catalytic site is highly conserved^74,75^. Targeting unique, non-catalytic (allosteric) surfaces offers a promising route to achieving specificity^76–78^, but a systematic method for identifying such sites has remained elusive.

**Figure 6.**
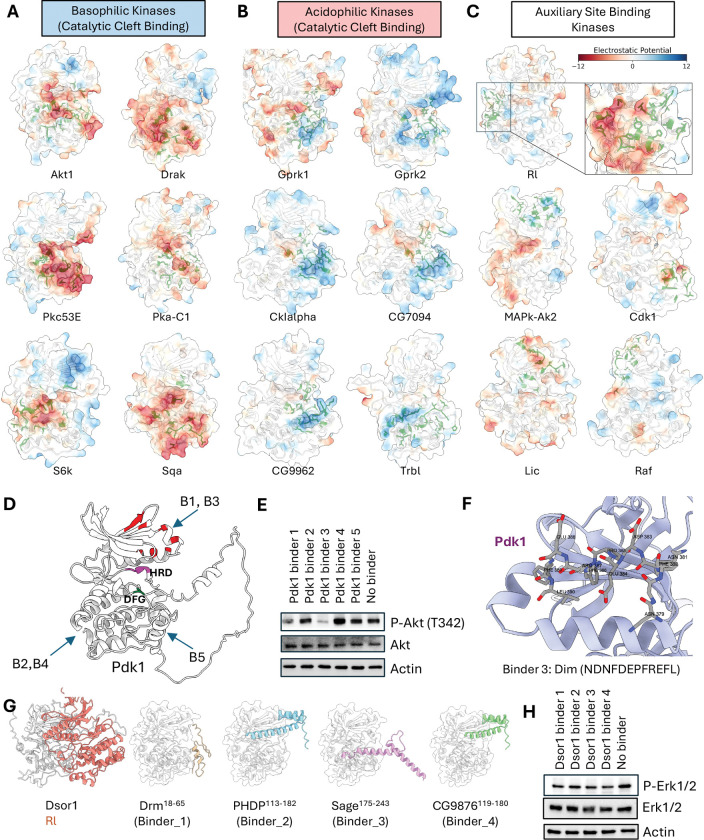
The kinome atlas identifies functionally relevant and inhibitable docking hotspots. (A–C) Principal modes of kinase partner recognition with electrostatic potential mapped to the kinase surface (red: acidic; blue: basic). (A) Basophilic kinases using the catalytic cleft. (B) Acidophilic kinases using the catalytic cleft. (C) Auxiliary site-binding kinases using non-catalytic (allosteric) surfaces. (D–F) Validation of a predicted allosteric hotspot on Pdk1. (D) Model of Pdk1 with interaction hotspots highlighted in red and predicted binding sites for five TF-derived fragments shown. Binders 1 & 3 target the allosteric PIF pocket. (E) Western blot showing that only Binders 1 & 3 inhibit downstream phosphorylation of Akt (at T342) in S2R+ cells. (F) Structural model of Binder 3 docked within the Pdk1 PIF pocket. (G–H) Validation of a predicted MAPK-docking site on Dsor1. (G) Models of the MAP2K Dsor1 and four TF-derived fragments predicted to inhibit Rl binding. (H) Western blot showing reduced phosphorylation of Rl (Erk1/2) in S2R+ cells upon expression of all four binder fragments.

Our atlas addresses this challenge by systematically mapping interaction hotspots across the entire kinase surface, revealing two principal strategies for partner recognition that are distinguished by their binding location. The first and most common strategy utilizes the catalytic cleft, which serves as the primary, electrostatically potent interface for kinases that prefer highly charged partners, such as the basophilic and acidophilic families (Fig. 6A, B; Supplementary Fig. 11A, B). In a second, contrasting strategy, kinases rely on auxiliary (allosteric) docking sites for partner engagement. This mode is employed by kinases that favor less-charged motifs, like the MAPK families (Fig. 6C; Supplementary Fig. 11C), but is also a noteworthy solution used by certain acidophilic kinases like CSNK2A1 and PLK1, demonstrating that diverse structural solutions for partner recognition exist (Supplementary Fig. 11D). This systematic classification of binding modes provides a powerful framework for identifying functionally critical—and potentially inhibitable—allosteric surfaces across the kinome.

To confirm that these predicted hotspots are functionally relevant and represent potential inhibitory targets, we experimentally validated two distinct sites. First, we focused on Pdk1. Its N-lobe contains the well-characterized PIF pocket, a docking site that is required for the phosphorylation and activation of key downstream substrates, including Akt1. Prior studies have shown that synthetic peptides like PIFtide can competitively inhibit this site, blocking downstream signaling and establishing it as a bona fide inhibitory target^79,80^. Consistent with this, our atlas identified the PIF pocket as the primary interaction hotspot on Pdk1 and predicted that fragments from several TFs would bind there. To test these predictions, we selected five TF-derived fragments: two targeting the PIF pocket hotspot, and three targeting other surfaces as controls (Fig. 6D). When expressed as BFP fusion proteins in *Drosophila* S2R+ cells, only the two fragments targeting the PIF pocket (Binders 1 and 3) effectively inhibited Pdk1’s downstream phosphorylation of Akt1, whereas the control fragments had no effect (Fig. 6E). Our structural models predict that these inhibitory peptides dock directly within the PIF pocket, providing a clear mechanistic basis for their competitive effect (Fig. 6F).

Second, we investigated a predicted hotspot on the MAP2K, Dsor1, required for the activation of its downstream MAPK, Rl. Among Dsor1-interacting TFs, we selected four high iLIS-scoring TF fragments. Our models predicted that these fragments would bind to the Dsor1 surface that engages Rl, suggesting they could act as competitive inhibitors (Fig. 6G). To test this, we expressed the fragments as BFP fusions in S2R+ cells. As predicted, all four fragments reduced phosphorylation of the downstream MAPK, Rl (Erk) (Fig. 6H). Notably, two of these inhibitory fragments were derived from homeodomain TFs (PHDP and CG9876), suggesting that in addition to being kinase targets, homeodomains may possibly, in certain contexts, also function as allosteric modulators of kinase activity.

Collectively, these results, where computational predictions for two distinct kinase families were supported by functional experiments, demonstrate the use of our atlas for the systematic discovery of functional, non-catalytic sites. We provide a comprehensive catalog of all interaction hotspots—encompassing both catalytic and putative allosteric surfaces—across the *Drosophila* and human kinomes (**Supplementary Data 5**), which can serve as a structural guide for developing a new generation of highly selective, allosteric kinase modulators.

### The kinome-TF atlas pinpoints regulators of Hnf4 and lipid homeostasis

**Figure 7.**
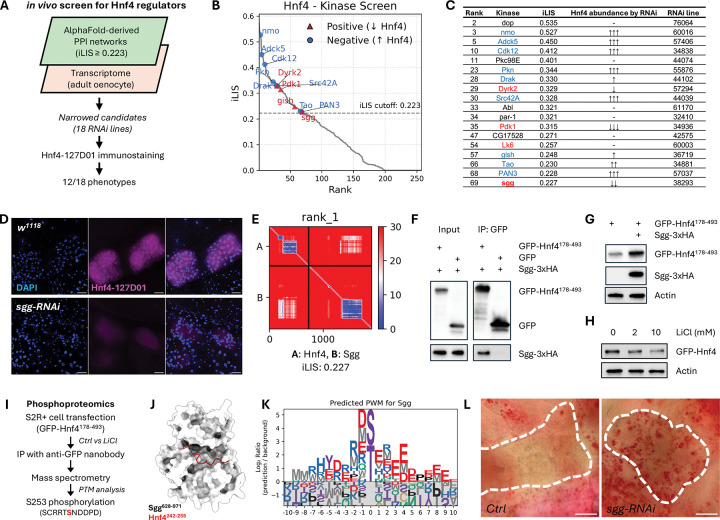
The kinome atlas identifies Sgg/GSK3 as a key regulator of Hnf4 stability and lipid homeostasis. (A) Schematic of the two-step *in vivo* screen to identify Hnf4 regulators. (B) Predicted iLIS scores for the 18 candidates from the *in silico* screen. Points are color-coded by their functional effect in the subsequent *in vivo* RNAi screen, and only candidates that produced a phenotype are labeled. (C) Summary of the *in vivo* RNAi screen results for the 18 candidates. *PromE-GAL4; tub-GAL80*^*ts*^ was used for oenocyte-specific RNAi. (D) Immunostaining of Hnf4 (magenta) in oenocytes from control (*w*^*111*^) and *sgg*-RNAi adults. DAPI (blue) stains nuclei. Scale bar, 20 μm. (E) Predicted Aligned Error (PAE) map of the Sgg-Hnf4 prediction. (F) Co-immunoprecipitation (co-IP) of Sgg-3xHA with GFP-Hnf4[178–493] from S2R+ cell lysates. (G) Western blot showing stabilization of the GFP-Hnf4[178–493] fragment upon Sgg-3xHA co-expression in S2R+ cells. (H) Western blot showing a reduced level of full-length GFP-Hnf4 in S2R+ cells after treatment with the GSK3 inhibitor LiCl. (I) Schematic of the phosphoproteomics workflow identifying Serine 253 (S253) as an Sgg-dependent phosphorylation site on Hnf4. (J) Structural model of the predicted Sgg-Hnf4 interface, highlighting the location of the S253-containing peptide (red). (K) Computationally derived PWM for Sgg from the fly kinome atlas. (L) Oil Red O staining of adult oenocytes, showing increased lipid accumulation (red) in *sgg*-RNAi compared to control. Knockdown was induced using the *PromE-GeneSwitch-Gal4* system; *sgg*-RNAi flies were treated with RU486 while control flies were not.

To test if our kinome-TF atlas could uncover physiologically relevant connections, we sought to identify novel regulators of *Drosophila* Hepatocyte Nuclear Factor 4 (Hnf4), a key transcription factor controlling lipid metabolism^81,82^. We implemented a two-step filtering strategy, first identifying kinases predicted to interact with Hnf4 with high confidence (iLIS≥0.223) and then intersecting this list with transcriptomic data from oenocytes to narrow down to 18 co-expressed kinases for functional validation (Fig. 7A). Oenocytes are a principal metabolic tissue in *Drosophila*, serving a role analogous to the mammalian liver. An adult oenocyte-specific RNAi screen of these candidates in endogenously NanoTagged Hnf4 flies (Hnf4–127D01)^83,84^ revealed that knockdown of 12 kinases (a 66% hit rate) altered Hnf4 protein abundance, underscoring the predictive power of our structure-guided approach (Fig. 7B, C; Supplementary Fig. 12).

Among the positive regulators identified in this screen was Shaggy (Sgg). We prioritized Sgg for in-depth validation because its mammalian ortholog, GSK3, is a key modulator of glycogen and lipid metabolism and is known to promote inflammation in pathologies like fatty liver disease^85,86^, making it a prime candidate for a conserved regulatory role. Confirming its importance, oenocyte-specific depletion of *sgg* led to a near-complete loss of Hnf4 protein *in vivo* (Fig. 7D), establishing Sgg as a critical regulator of Hnf4.

Guided by the structural prediction (Fig. 7E), we proceeded to dissect the mechanism of this interaction. We first validated a physical interaction in S2R+ cells, showing that Sgg co-immunoprecipitated with a GFP-tagged Hnf4 fragment (Hnf4[178–493]) that contained the local interaction area with Sgg (Fig. 7F). Next, we confirmed that Sgg’s kinase activity is required for Hnf4 regulation. While Sgg overexpression stabilized the Hnf4[178–493] fragment (Fig. 7G), inhibiting GSK3 with lithium chloride (LiCl) reduced full-length Hnf4 protein levels (Fig. 7H).

To identify the phosphorylation site, we performed phosphoproteomics on the immunoprecipitated GFP-Hnf4[178–493] fragment. This analysis identified Serine 253 (S253) as a site whose phosphorylation is dependent on Sgg/GSK3 activity, as its signal was rendered undetectable by LiCl treatment (Fig. 7I). Typically, GSK3 activity is priming-dependent, requiring a prior phosphorylation event by a different ‘priming kinase’ on a serine or threonine located at the +4 position relative to the target site, creating the canonical S/T-x-x-x-pS/pT docking motif for Sgg/GSK3^87–89^. The sequence surrounding S253 does not conform to this canonical motif. However, priming-independent mechanisms, in which GSK3 can phosphorylate a substrate without a priming event, have been documented for other substrates such as Myc, Jun, and Tau^90,91,89^.

Consistent with such a mechanism, the sequence C-terminal to S253 features an acidic patch (NDDPD). Our structural model predicts that this Hnf4 peptide docks near the catalytic site of Sgg (Fig. 7J). This principle is corroborated by our computationally derived PWM for Sgg, which reveals a strong general preference for acidic residues downstream of the phospho-acceptor (Fig. 7K). These converging lines of evidence suggest a mechanism wherein Sgg directly phosphorylates Hnf4 at S253 to control its stability.

Finally, to determine the physiological consequence of this Sgg-Hnf4 regulatory axis, we depleted *sgg* specifically in oenocytes. This manipulation induced marked lipid accumulation, a steatosis-like phenotype that phenocopies *Hnf4* loss (Fig. 7L)^81^. Together, these data validate Sgg as a critical kinase that stabilizes Hnf4 via a priming-independent phosphorylation at S253, and demonstrates the use of our atlas for discovering novel kinases that control organismal metabolism *in vivo*.

## Discussion

Here, we present the first systematic, structure-guided kinase–TF interactome atlas, contributing to our understanding of a longstanding challenge in cell signaling. By pairing AlphaFold-Multimer with our iLIS framework, we address a key limitation of existing metrics in recognizing interactions mediated by intrinsically disordered regions. The robustness of the resulting atlas is validated against multiple benchmarks, where it recovers known physical interactions from BioGRID and corresponds with functional co-dependencies from DepMap. This work therefore provides a map of the kinome-TF interactome that captures the flexible, transient interactions underpinning signaling specificity.

A key advance of this atlas is its ability to move beyond binary interaction mapping to reveal how and where interactions occur, resolving specific interfaces that have remained elusive in large-scale screens. The atlas provides residue-level structural insight into partner recognition, exemplified by our binding-defined classification of the kinome, the discovery of a putative conserved docking mechanism for homeodomain TFs, and the systematic mapping of allosteric hotspots. The HD-TF analysis, supported by extensive computational modeling and *in silico* mutagenesis, proposes a detailed hypothesis for how these developmental regulators are engaged by upstream pathways.

We demonstrate the atlas’s power as a hypothesis-generation approach by validating in vivo a high-confidence predicted interaction between Sgg and Hnf4. Our platform successfully guided the identification of a specific, Sgg-dependent phosphorylation site at Serine 253. The biochemical features of this site were consistent with the binding preferences for Sgg derived from our atlas, elucidating a non-canonical, priming-independent phosphorylation mechanism that controls lipid metabolism *in vivo*. This mechanism is consistent with studies of other GSK3 substrates, where acidic residues can substitute for a priming phosphate, although often with reduced kinetic efficiency^92^. The physical interaction we observed between Sgg and Hnf4 may compensate for this potential inefficiency through induced proximity, increasing the effective local concentration of enzyme and substrate to drive physiologically relevant phosphorylation *in vivo*.

This structural atlas lays the groundwork for advances in drug development. While achieving selectivity against the conserved ATP-binding pocket is a persistent challenge^93–95^, our atlas provides a detailed resource for targeting unique, non-catalytic (allosteric) surfaces. With recent advances in designing bespoke protein, peptide, and small-molecule binders for specific target proteins^96–105^, the primary hurdle is shifting from how to target a surface to which surface provides the desired therapeutic effect. Our work provides a catalog of these surfaces across human and fly kinases, paving the way for the development of selective, non-ATP-site kinase modulators.

Our approach has some inherent limitations. Its accuracy is fundamentally dependent on the underlying AlphaFold-Multimer predictions, and our models analyze binary interactions in isolation, without crucial cellular context like co-factors or subcellular compartmentalization. A key limitation is the absence of post-translational modifications in our models, such as the priming phosphorylation events required by kinases like GSK3. While this prevents the detection of strictly priming-dependent interactions, it creates a productive bias that enriches for alternative, priming-independent recognition mechanisms. As demonstrated with the Sgg-Hnf4 interaction, our platform successfully identifies cases where the kinase recognizes a patch of acidic residues that functionally mimics a priming phosphate, positioning the atlas as a unique resource for exploring these non-canonical modes of kinase regulation. Future integration with large-scale phosphoproteomic datasets will be a powerful next step to systematically distinguish between priming-independent docking and true enzymatic substrates.

Beyond the limitations of the structural models, it is critical to distinguish a predicted docking event from a functional enzymatic one, as high-confidence binding does not guarantee phosphotransfer. Well-characterized pseudosubstrates, such as the Protein Kinase Inhibitor (PKI) peptide, exploit this principle by binding a kinase’s active site without being phosphorylated to act as inhibitors^106–108^. Consequently, some high-confidence interactions in our atlas may represent inhibitory or scaffolding relationships rather than enzymatic substrate modifications. Finally, because the atlas is based on a defined confidence threshold, it may exclude genuine low-affinity interactions, and the direct extrapolation of specific findings to other systems requires further system-specific validation.

Future directions include integration with quantitative phosphoproteomics, transcriptomic expression profiles, and genetic interaction maps to refine functional annotation. Extending AFM + iLIS to ubiquitin ligases, phosphatases, and additional protein families will test generality and accelerate discovery of non-canonical regulatory interfaces. The newly identified docking sites—particularly within IDRs—offer untapped opportunities for allosteric and degradation-tag inhibitor design, complementing classical active site-targeting strategies.

By charting both classical and unconventional kinase docking landscapes at unprecedented scale, our atlas empowers hypothesis-driven exploration of signaling regulation and provides a versatile platform for the rational development of next-generation kinase modulators. Ultimately, by providing a structural blueprint of the kinome, this work demonstrates how predictive structural biology is evolving from a tool for understanding individual proteins into an approach for the rational modulation of entire cellular networks.

## Methods

### AlphaFold-Multimer prediction

For AFM predictions, we employed LocalColabFold v.1.5.5^109^, which integrates AlphaFold-Multimer (AFM) v.2.3.1. MSAs were generated using colabfold_search against the UniRef30 (version 2202) and mmseqs2/14–7e284 structural databases. All computations were performed on the Harvard O2 high-performance computing cluster and the Longwood high-performance compute cluster using a variety of NVIDIA GPUs (TeslaV100, RTX6000, RTX8000, A40, A100, L40S, and H100). Each prediction generated five models with five recycling. Interaction confidence was assessed using the integrated Local Interaction Score (iLIS), with a high-confidence threshold of iLIS ≥ 0.223 established at a 10% FDR based on a comprehensive benchmark against curated positive and control reference sets (see Supplementary Text 1–4). iLIS analysis code and benchmarking thresholds based on literature-based datasets are available at https://github.com/flyark/AFM-LIS.

### Interaction fingerprint clustering

To group a target protein’s interaction partners based on their binding patterns, we developed a pipeline to generate and cluster “interaction fingerprints.” For each target protein, a set of high-confidence binding partners (iLIS≥0.223) was used as input.

First, a binary contact matrix was constructed where each row represented an interacting partner and each column represented a residue position on the target protein. A value of 1 was assigned if a partner made contact with a specific target protein residue (as defined by the contact-filtered Local Interaction Residues, or cLIRs), and 0 otherwise.

These binary fingerprints were then subjected to agglomerative hierarchical clustering using the SciPy library^110^. The similarity between fingerprints was measured using the cosine distance, and clusters were merged using the average linkage (UPGMA) algorithm. To determine the optimal number of clusters for each target protein automatically, we tested a range of cluster numbers (from 2 to 30) and calculated the Silhouette Score for each partition using the scikit-learn library^111^. This score measures the quality of the clustering, and the number of clusters that maximized this score was chosen as the optimal grouping. The resulting clusters, representing distinct binding modes, were visualized using a clustermap (seaborn.clustermap)^112^ where partners are ordered by the clustering dendrogram.

### Binding-defined kinase tree generation

The binding-defined kinase tree was generated in R from a kinase co-occurrence matrix derived from a stability analysis of 10,800 clustering runs (see Supplementary Text 7). The similarity matrix was converted to a distance matrix and subjected to agglomerative hierarchical clustering (complete linkage). The final clusters were defined using the Dynamic Tree Cutting algorithm (cutreeDynamic R package)^113^. The resulting dendrogram was visualized using the ggtree package^114^.

### Sequence-based kinase tree generation

To generate a phylogenetic tree based on sequence homology (as shown in Figure 4D), kinase domain sequences from *Drosophila melanogaster* were obtained, with family classifications (e.g., AGC, CMGC) based on annotations from KinBase^51^ and FlyBase^115^. The analysis was performed in R. First, a multiple sequence alignment of the kinase domain amino acid sequences was generated using the Muscle algorithm^116^, implemented in the msa package^117^. The resulting alignment was then used to calculate a maximum likelihood distance matrix using the JTT (Jones-Taylor-Thornton) model of amino acid substitution ^118^ via the phangorn package^119^. From this distance matrix, a phylogenetic tree was constructed using the Neighbor-Joining algorithm^120^, as implemented in the ape package^121^. Finally, all kinases belonging to the “Atypical” class were pruned from the tree to focus the visualization on the major kinase families. The final phylogeny was plotted as an unrooted tree using the ggtree package^114^.

### Validation of predicted protein-protein interactions using external datasets

To validate the predicted human kinase-transcription factor interactions, the atlas was cross-referenced against two independent, large-scale datasets. For physical and genetic Interactions, known protein-protein and genetic interactions were sourced from the BioGRID database (Version 4.4.245)^45^. The datasets for *Homo sapiens* and *Drosophila melanogaster* were downloaded and integrated for comparison with our predictions. To identify functional co-dependency, functional relationships were assessed using data from the Cancer Dependency Map (DepMap, Public 25Q2)^46^. High-confidence structural predictions from our atlas were compared against the top 100 functionally co-dependent gene partners (ranked by absolute correlation) for each corresponding kinase or transcription factor in the DepMap CRISPR screen dataset.

### Comparison with experimentally derived PWMs

Computationally derived Position Weight Matrices (PWMs) were compared to experimentally determined motifs from Johnson et al., 2023^4^. The normalized and scaled PSSM data (ser_thr_all_norm_scaled_matrice), raw matrix data (ser_thr_all_raw_matrices), and kinase metadata were extracted from the supplementary materials of the publication for direct comparison and visualization. A mapping between the gene names used in our study and the “Matrix_name” identifiers was created to ensure accurate correspondence between predicted and experimental motifs. Sequence logos for human PWMs were generated using logomaker pagage in R^122^.

### Data analysis and visualization

Statistical analyses, including Receiver Operating Characteristic (ROC) curve analysis, were performed using the scikit-learn^111^ and SciPy^110^ libraries in Python. Figures were generated using Matplotlib^123^, Seaborn^112^, and logomaker^124^ in Python, or the ggtree package^114^ in R. Protein structures and electrostatic potentials were visualized in UCSF ChimeraX v1.9^125^. Electrostatic surfaces were calculated in ChimeraX using the coulomb command with a rdbu-7 palette and a charge range of −12 to 12.

### Cell culture and transfection

*Drosophila* S2R+ cells (DGRC, Cat# 150) were maintained at 25°C in Schneider’s Drosophila Medium (Thermo Fisher Scientific, Cat# 21720–024) supplemented with 10% Fetal Bovine Serum (Sigma, Cat# A3912) and 50 U/ml Penicillin-Streptomycin (Thermo Fisher Scientific, Cat# 15070–063). For all experiments, cells were transfected in 6-well plates using the Effectene Transfection Reagent (Qiagen, Cat# 301427) according to the manufacturer’s protocol.

### Cloning and expression

Expression vectors were constructed using cDNA from the FlyBi ORF collection ^33^ or synthetic DNA fragments (IDT or Twist Bioscience). For validation of allosteric hotspots, transcription factor fragments identified in the AFM screens against Pdk1 and Dsor1 were synthesized and cloned into the pMT vector with N-terminus 127D01 tag and C-terminus BFP tag (pMT-127D01-Binder-BFP).

**Table:** 

Binders	Binder fragment sequences
Pdk1_Binder_1	SSSAWRPW
Pdk1_Binder_2	ASRWLTEEVFKLIEIVQRDEAIYNPRHKYYFCRPYVENFWREVDLKLEKNPGASLAKWTNLRISFRREYTNYLEEKVPPCWSYFDRMFFLHPYLRKKHQQP
Pdk1_Binder_3	NDNFDEPFREFL
Pdk1_Binder_4	SWSRAVFSNLQRKGLEIQFQQQKYITKPDRRKLAARLNLTDAQVKVWFQNRRMKWRHTRENLKSGQE
Pdk1_Binder_5	FTLTAQNVCAKCNISFRMTSDLVYHMRSHHKSEVA
Dsor1_Binder_1	RKMRPKCEFICKYCQRRFTKPYNLMIHERTHKSPEITYSCEVCGKYFK
Dsor1_Binder_2	RRIRTTFTSNQLNELEKIFLETHYPDIYTREEIASKLHLTEARVQVWFQNRRAKFRKQERHAIYIMKDKS
Dsor1_Binder_3	QMEKDYRRTACDRERTRMRDMNRAFDLLRSKLPISKPNGKKYSKIESLRIAINYINHLQAMLRESSVGQ
Dsor1_Binder_4	RNRTTFSSAQLTALEKVFERTHYPDAFVREELATKVHLSEARVQVWFQNRRAKFRRNERSVG

For the Hnf4 analysis, DNA fragments for full-length Hnf4 and the Hnf4[178–493] fragment were synthesized and cloned into a pMT vector for N-terminal GFP tagging (pMT-CMP-v1-GFP-Hnf4-full and pMT-CMP-v1-GFP-Hnf4-[178–493]). The Sgg FlyBi clone was cloned into the pAWH vector (DGRC, Cat# 1096) for C-terminal 3xHA tagging (pAWH-sgg).

Expression from vectors containing the pMT promoter was induced with 500 μM copper sulfate (CuSO4). Other constructs were expressed constitutively from pAct promoter.

### Co-immunoprecipitation and western blotting

For co-IP experiments, transfected cells were harvested, washed twice in PBS, and lysed on ice in CHAPS lysis buffer (Boston BioProducts, Cat# BP-114) supplemented with 2x protease and phosphatase inhibitor cocktails (Thermo Fisher Scientific, Cat# A32961). Cell lysates were cleared by centrifugation at 20,000 × g for 10 minutes at 4°C.

Cleared lysates were incubated with nanobodies coupled to magnetic beads overnight at 4°C with rotation. Proteins tagged with GFP were immunoprecipitated using an in-house purified anti-GFP nanobody (NbGFP-ALFA-His) conjugated to ALFA-tag magnetic resin (NanoTag Biotechnologies, Cat# N1512). Proteins tagged with mCherry were immunoprecipitated using mCherry-Trap magnetic resin (ChromoTek, Cat# rtma). Following incubation and washes, bound proteins were eluted, separated by SDS-PAGE (Bio-Rad, Cat# 4568096), and analyzed by western blot.

Primary antibodies used were: rabbit anti-GFP (Invitrogen, Cat# A-6455), rat anti-HA (Roche, Cat# 11867423001), rabbit anti-P-Akt (T342) (PhosphoSolutions, Cat# p104–342), rabbit anti-Akt (Cell Signaling Technology, Cat# 9272S), rabbit anti-P-ERK1/2 (Cell Signaling Technology, Cat# 4370S), and rabbit anti-ERK1/2 (Cell Signaling Technology, Cat# 4695S). An anti-Actin-Rhodamine antibody (Bio-Rad, Cat# 12004163) was used as a loading control. HRP-conjugated anti-rabbit (Jackson ImmunoResearch, Cat# 111–035-003) and anti-rat secondary antibodies (Jackson ImmunoResearch, Cat# 112–035-062) were used for detection with Pico Plus (Thermo Scientific, Cat# 34580) or Femto ECL chemiluminescent substrates (Thermo Scientific, Cat# 34095). Images were acquired using a Bio-Rad ChemiDoc MP imaging system.

### Lithium chloride treatment

To assess protein stability, S2R+ cells were transfected with a pMT-CMP-v1-GFP-Hnf4-full plasmid. Following a 24-hour induction with 500 μM CuSO4, the GSK3 inhibitor lithium chloride (LiCl) was added to the media for an additional 16 hours. Cells were then collected and processed for western blot analysis.

### Phosphoproteomics

S2R+ cells were transfected with a Hnf4[178–493] expression vector (pMT-CMP-v1-GFP-Hnf4-[178–493]). After induction with 500 μM CuSO4, cells were treated with either a control buffer or 10 mM LiCl for 16 hours. The GFP-Hnf4[178–493] fragment was immunoprecipitated from cleared cell lysates using anti-GFP nanobody conjugated to ALFA-tag magnetic resin. Eluates by ALFA peptide were separated by SDS-PAGE, and the gel bands corresponding to the molecular weight of the Hnf4 fragment were excised and submitted for phosphorylation site mapping in the Mass Spectrometry Facility at Beth Israel Deaconess Medical Center.

### *Drosophila* stocks and husbandry

All experiments were conducted using female flies due to their larger oenocyte size, which facilitated dissection. For temperature-sensitive experiments, fly crosses were set up and maintained under a 12:12 hour light:dark cycle at 18°C. Crosses were discarded after 5 days to maintain consistent population density. Following eclosion, adult flies were collected and kept at 18°C for 2–3 days. *w; Hnf4–127D01; PromE-GAL4, Tub-GAL80*^*TS*^ flies were crossed with following Bloomington Drosophila Stock Center RNAi lines: *par-1* (#32410), *gish* (#36719), *sgg* (#38293), *Lk6* (#60003), *Pdk1* (#34936), *Cdk12* (#34838), *nmo* (#60016), *Tao* (#34881), *Drak* (#44102), *Abl* (#61170), *Dyrk2* (#57294), *Pkc98E* (#44074), *Src42A* (#44039), *PAN3* (#57037), *dop* (#76064), *Pkn* (#55876), *CG17528* (#42575), and *Adck5* (#57406).

### In vivo immunostaining

For nanobody immunostaining, adult fly abdomens were dissected in PBS at room temperature, the fat body was removed, and tissues were fixed in 4% paraformaldehyde in PBS for 30 min. Samples were washed 3 × 5 min in PBST (PBS + 0.1% Triton X-100) and blocked in PBST + 5% normal goat serum (NGS) for 1 h. The anti-ALFA nanobody Nb127D01-ALFA (0.2 mg/mL stock) was diluted 1:500 in PBST + 5% NGS and applied for 1.5 h at room temperature with gentle agitation, followed by 3 × 5 min washes in PBST. Secondary antibody Alexa Fluor 594 anti-Alpaca (Jackson ImmunoResearch, Cat# 128–585-232) was diluted 1:400 in PBST + 5% NGS and incubated with samples for 1 h at room temperature. After PBST washes and DAPI counterstaining, tissues were mounted in VECTASHIELD Antifade Mounding Medium (Vector Laboratories, Cat# H-1000–10) and imaged on an Olympus VS200 slide scanner or a Zeiss Axio Observer Z1 confocal microscope.

### Oil Red O staining

Oil Red O staining was performed as previously described(Gutierrez *et al.*, 2007) with minor modifications. Adult flies were dissected in ice-cold PBS, and fat bodies were removed by gentle aspiration (“liposuction”). Tissues were fixed in 4% paraformaldehyde for 20 min at room temperature, rinsed twice in distilled water, and stained with freshly prepared Oil Red O. For the stain, Oil Red O (Sigma-Aldrich, Cat# O0625) was dissolved in isopropanol to 0.1% (w/v) to prepare a stock solution; the working solution was made immediately before use by mixing 3 mL of stock with 2 mL of distilled water and filtering through a 0.2-μm syringe filter. Samples were incubated in the working solution for 25 min, briefly rinsed in PBS, and mounted in VECTASHIELD medium for imaging. Images were acquired on an Olympus VS200 slide scanner equipped with a color camera (2448 × 2048 pixels; 3.45-μm pixel size).

## Supplementary Material

Supplement 1

Supplement 2

Supplement 3

Supplement 4

Supplement 5

Supplement 6

Supplement 7

Supplement 8

Supplement 9

Supplement 10

Supplement 11

Supplement 12

Supplement 13

Supplement 14

## Data Availability

The complete fly kinase–TF atlas, including iLIS scores and models, is available for interactive exploration at the FlyPredictome portal (https://www.flyrnai.org/tools/fly_predictome). All supplementary datasets generated in this study, including the residue-level interaction data (**Supplementary Data 1** and **3**), the complete PWMs for the human and fly screens (**Supplementary Data 2** and **4**), the comprehensive catalog of interaction hotspots (**Supplementary Data 5**), and the multi-page Supplementary Figure 8 (containing sequence logos for all fly kinase clusters) have been deposited in the Zenodo repository and are publicly available under DOI: 10.5281/zenodo.17314335.
