# Supplementary figures and images for: A Structure-Guided Kinase–Transcription Factor Interactome Atlas Reveals Docking Landscapes of the Kinome
Source: bioRxiv. 2025 Nov 25:2025.10.10.681672. Preprint. [Version 4] doi: 10.1101/2025.10.10.681672 (PMC12632555; doi:10.1101/2025.10.10.681672)

# Supplementary Figure 1

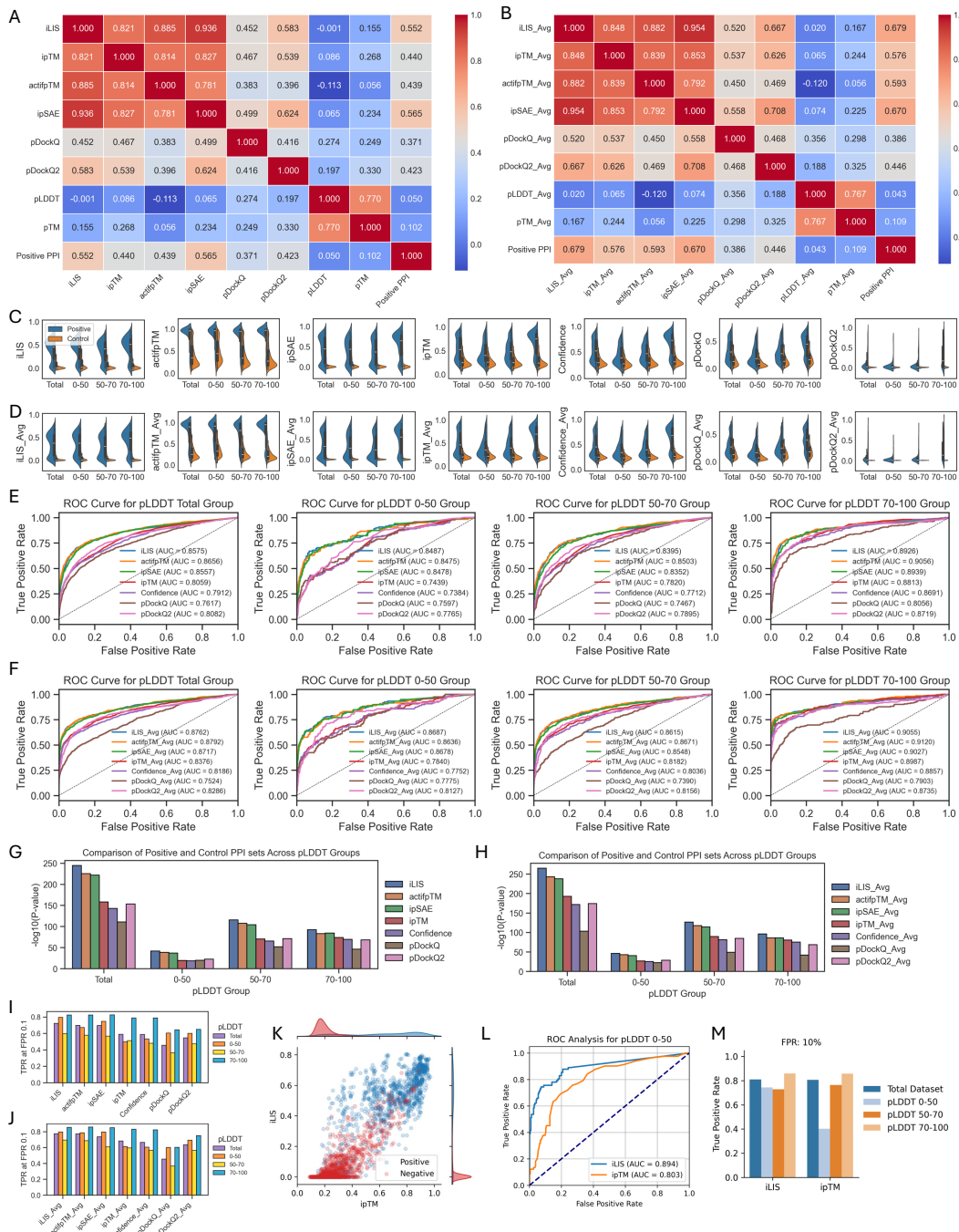

Supplement: Supplement 1 [file media-1.pdf]

# Supplementary Figure 3

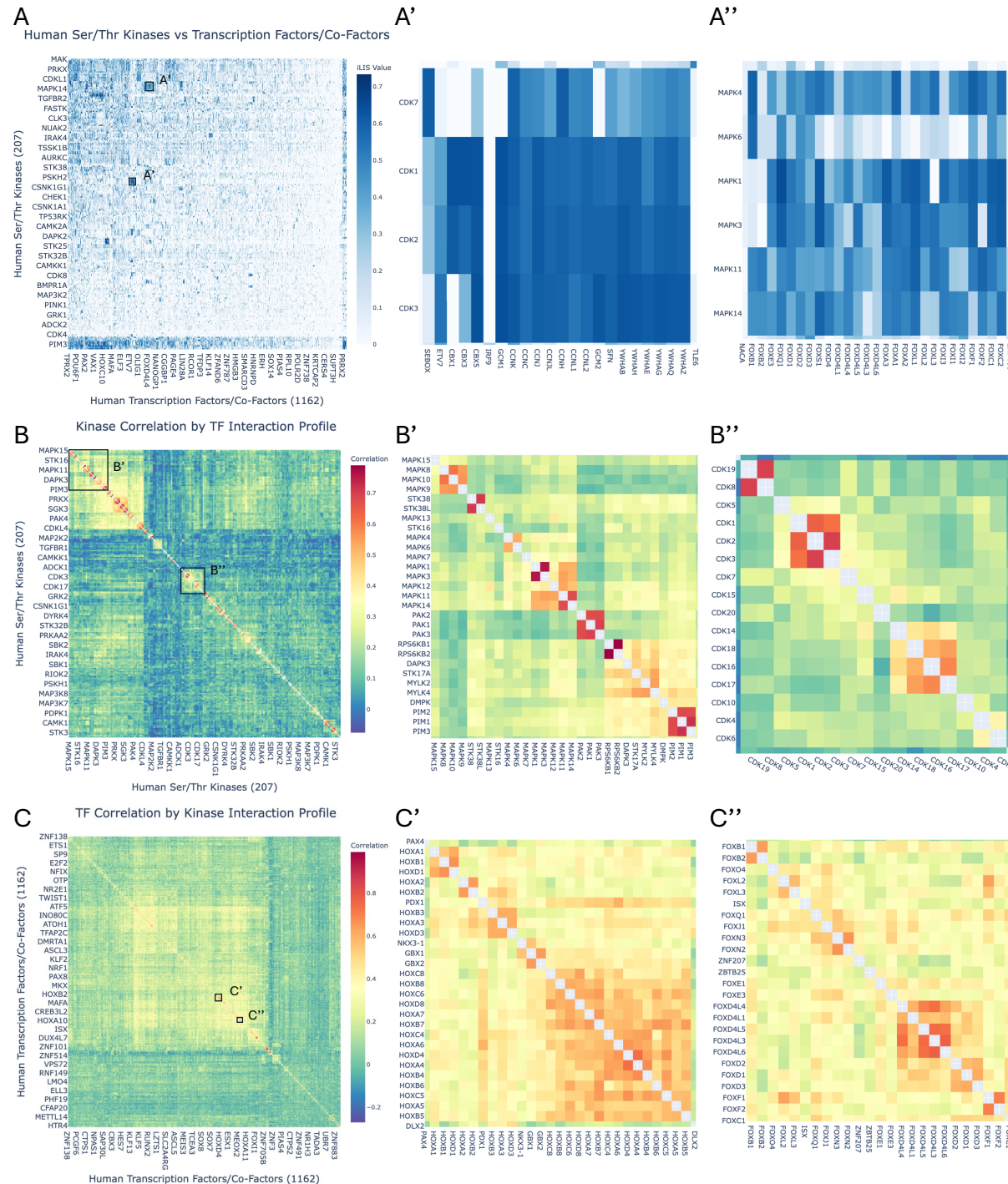

Supplement: Supplement 3 [file media-3.pdf]

# Supplementary Figure 4

A

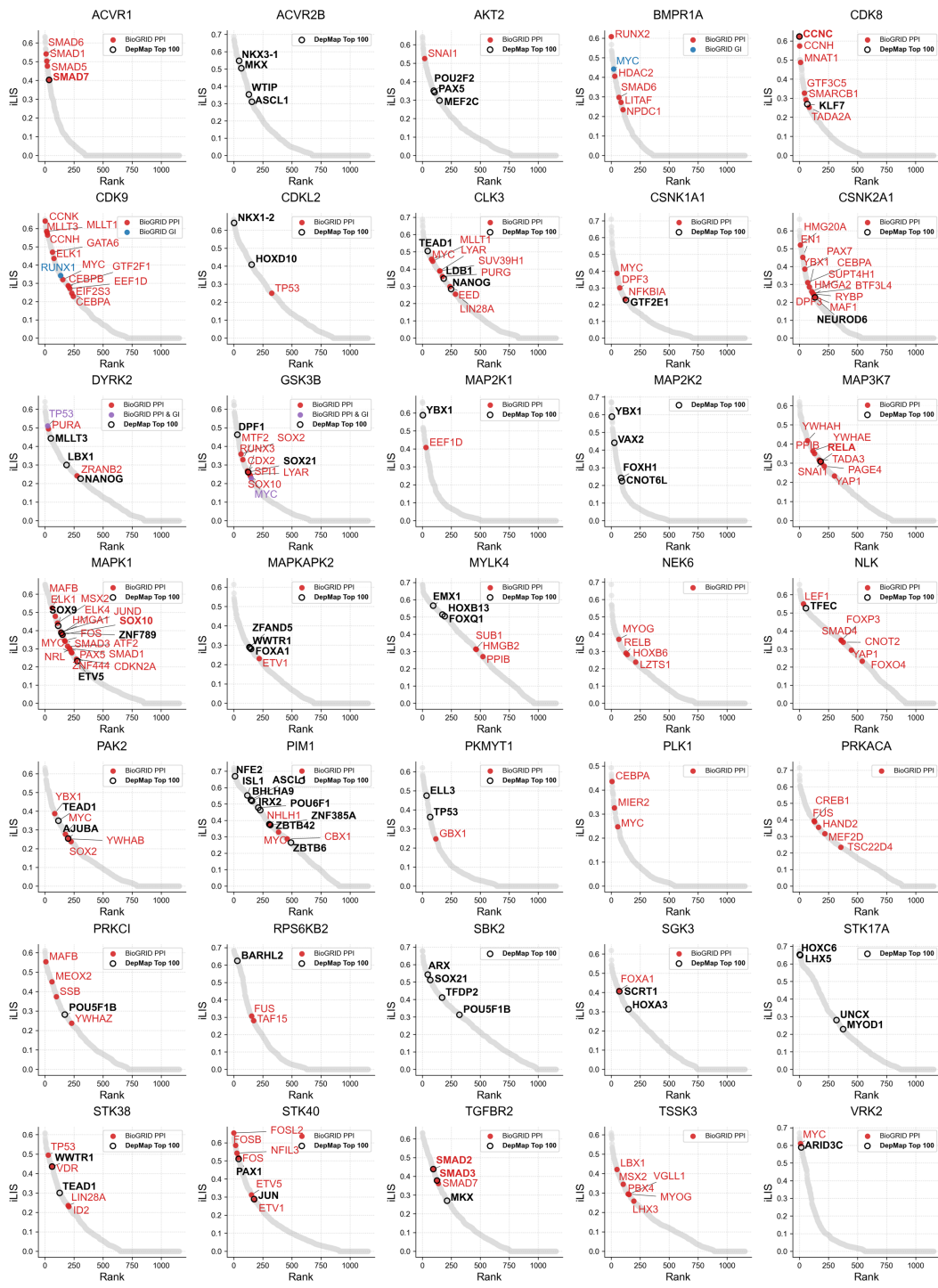

Supplement: Supplement 4 [file media-4.pdf]

# Supplementary Figure 4

B

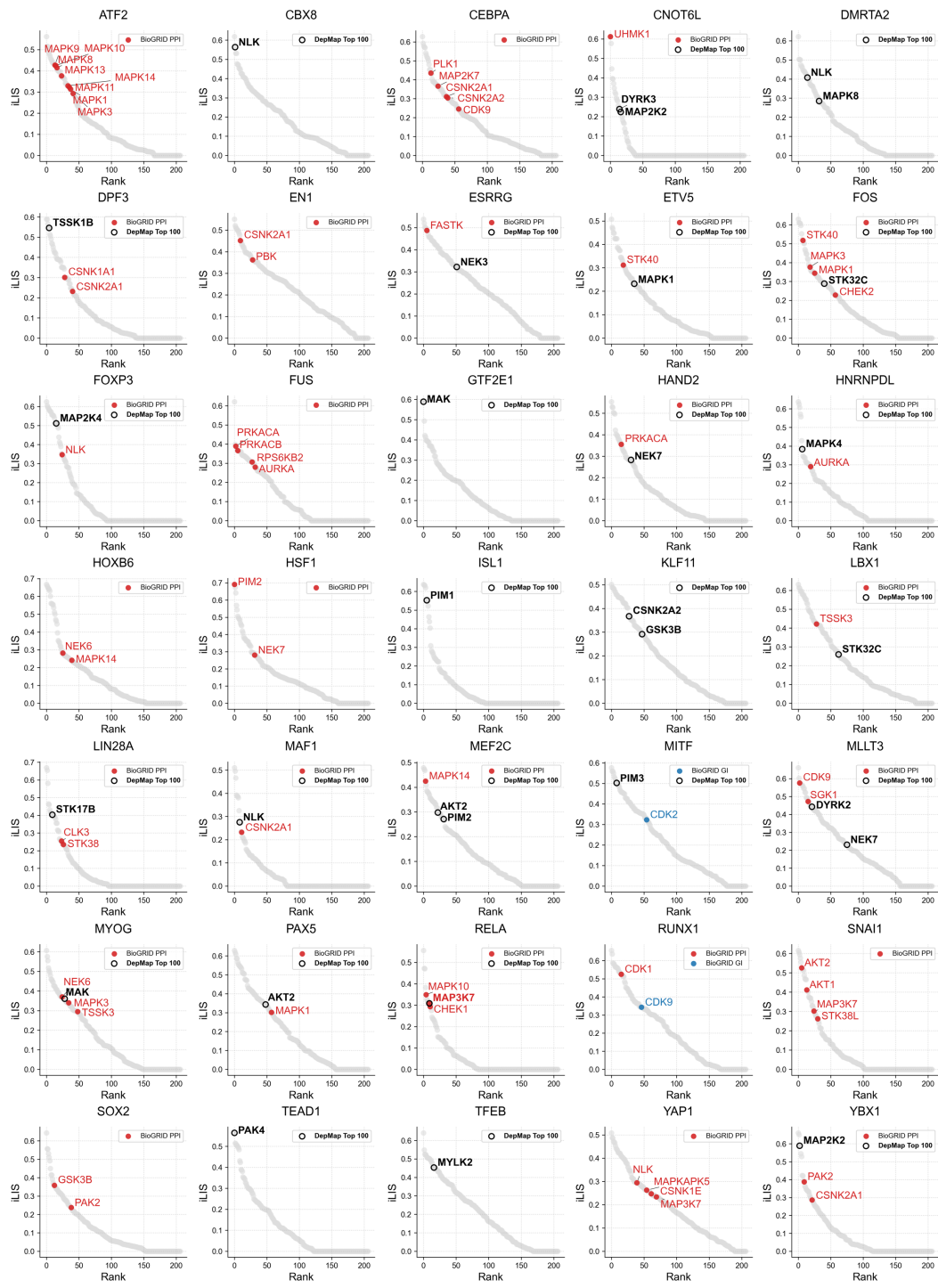

Supplement: Supplement 5 [file media-5.pdf]

# Supplementary Figure 5

A

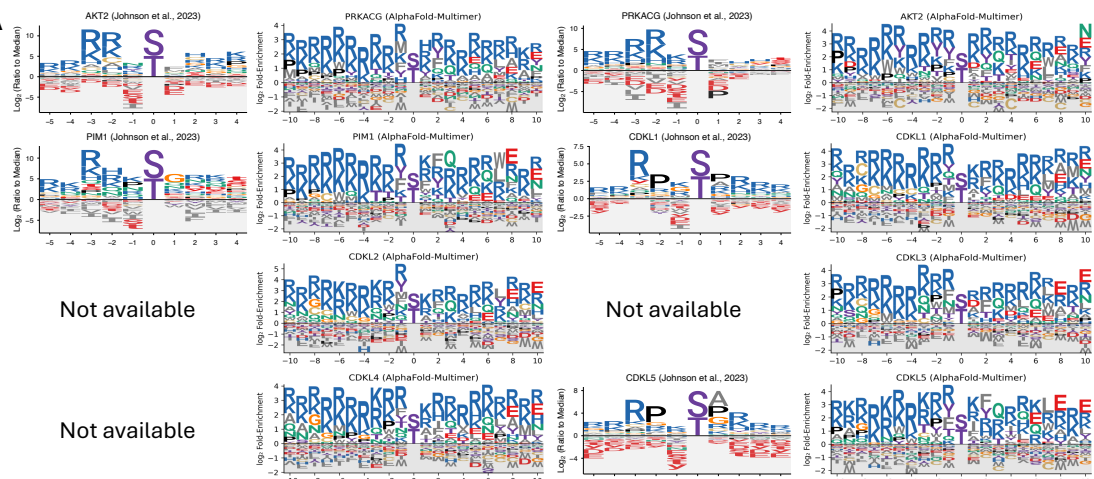

B

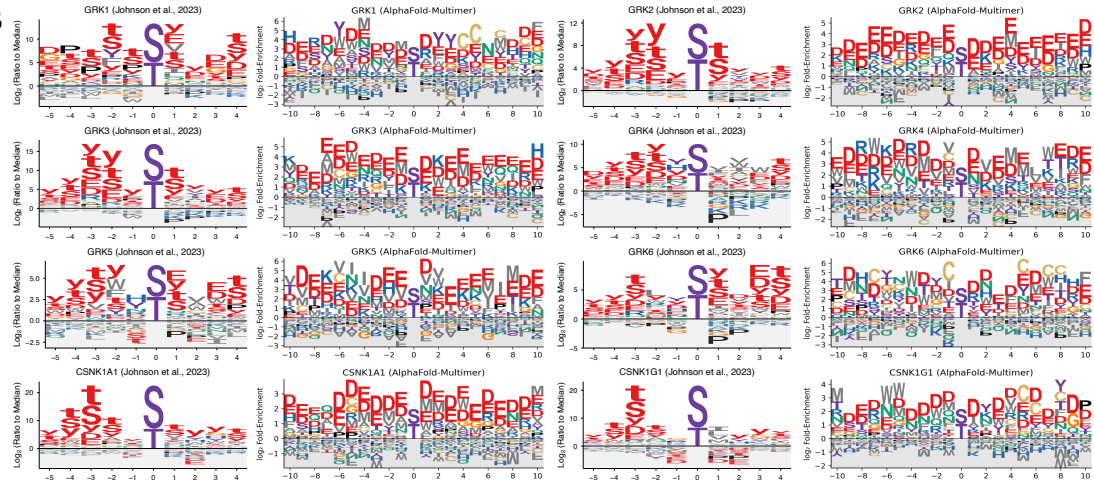

C

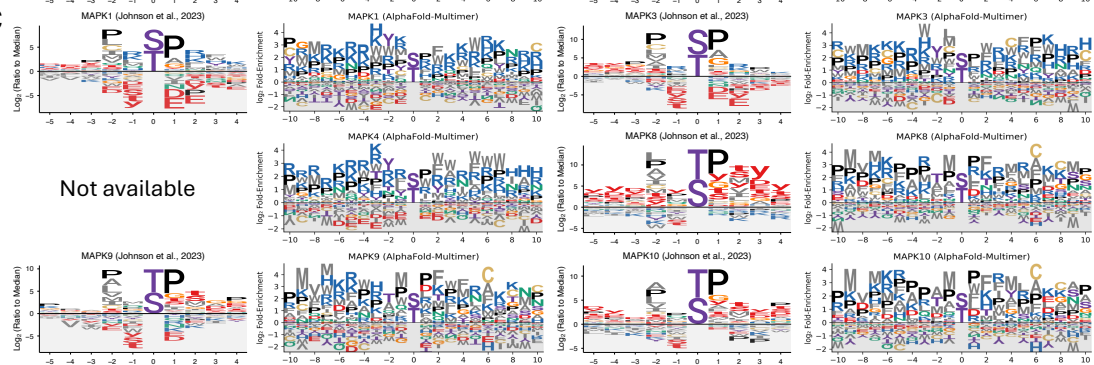

Supplement: Supplement 6 [file media-6.pdf]

# Supplementary Figure 6

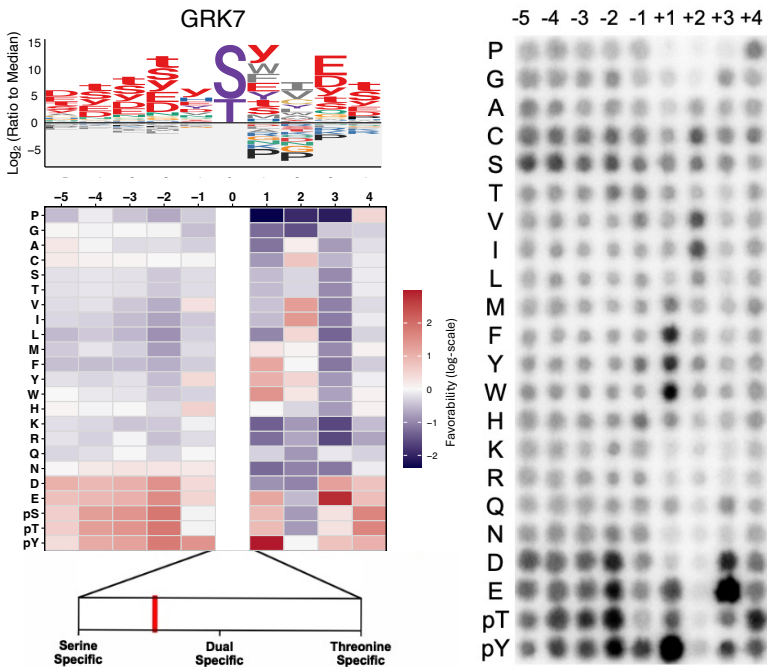

Supplement: Supplement 7 [file media-7.pdf]

# Supplementary Figure 7

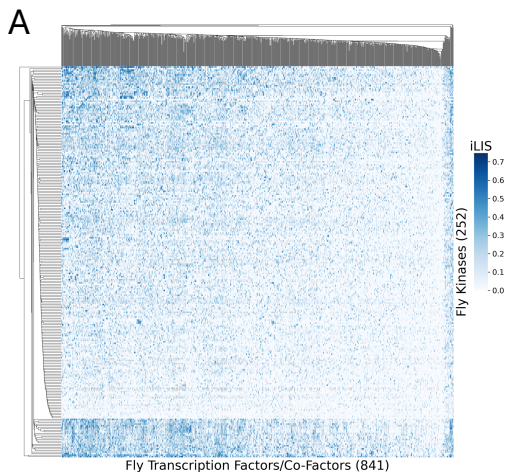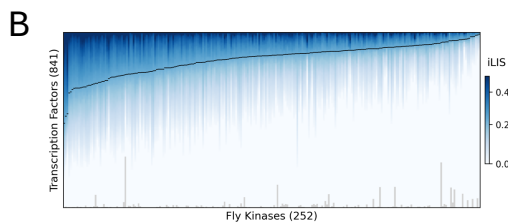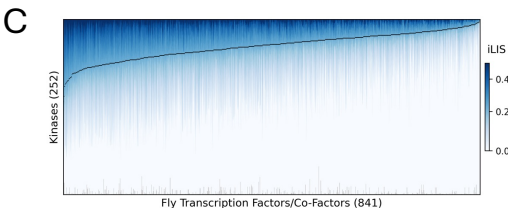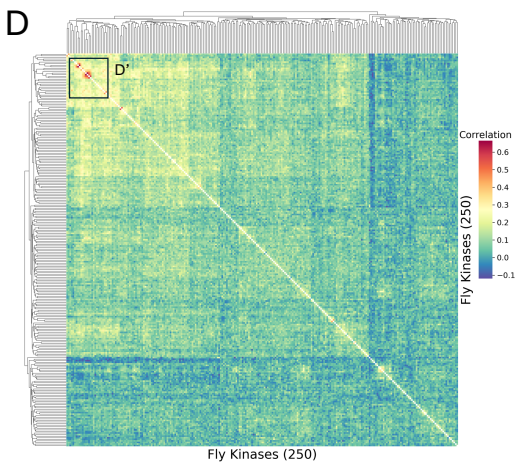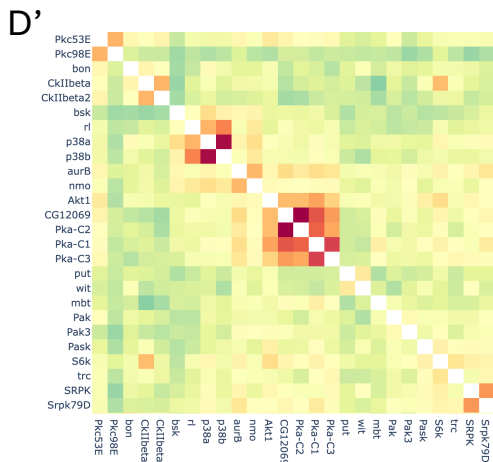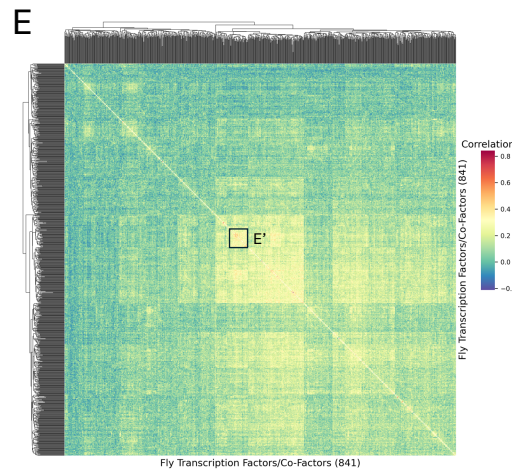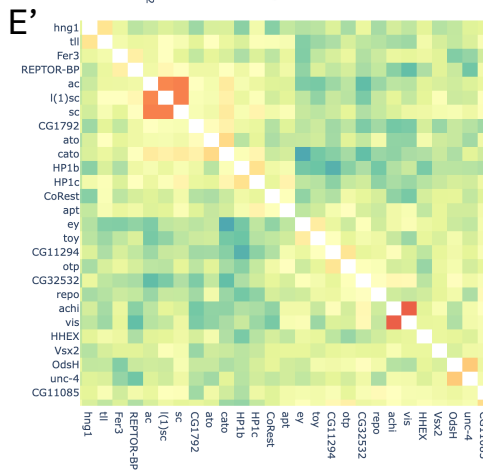

Supplement: Supplement 8 [file media-8.pdf]

Supplementary Figure 9

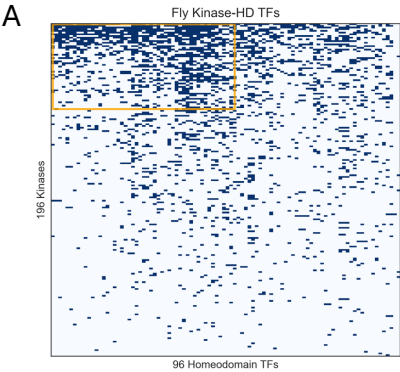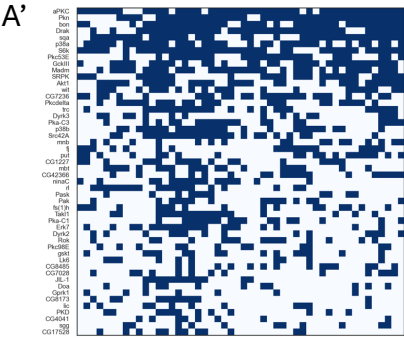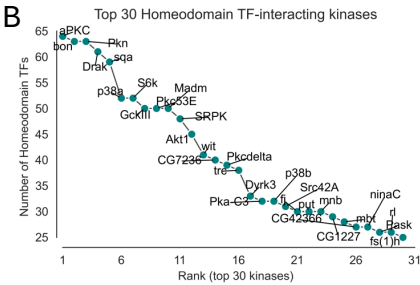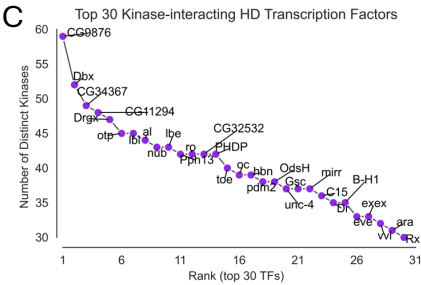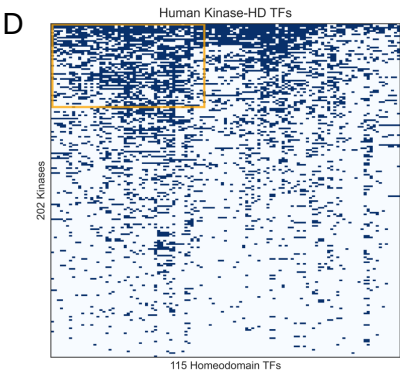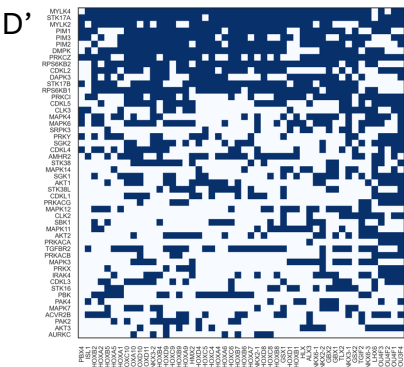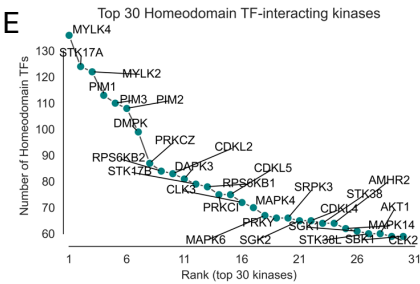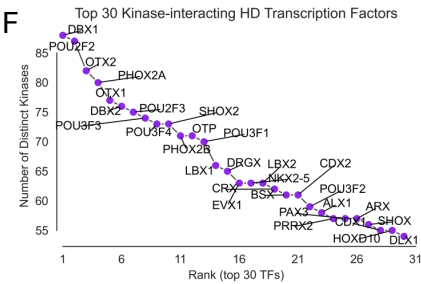

Supplement: Supplement 10 [file media-10.pdf]

# Supplementary Figure 10

A

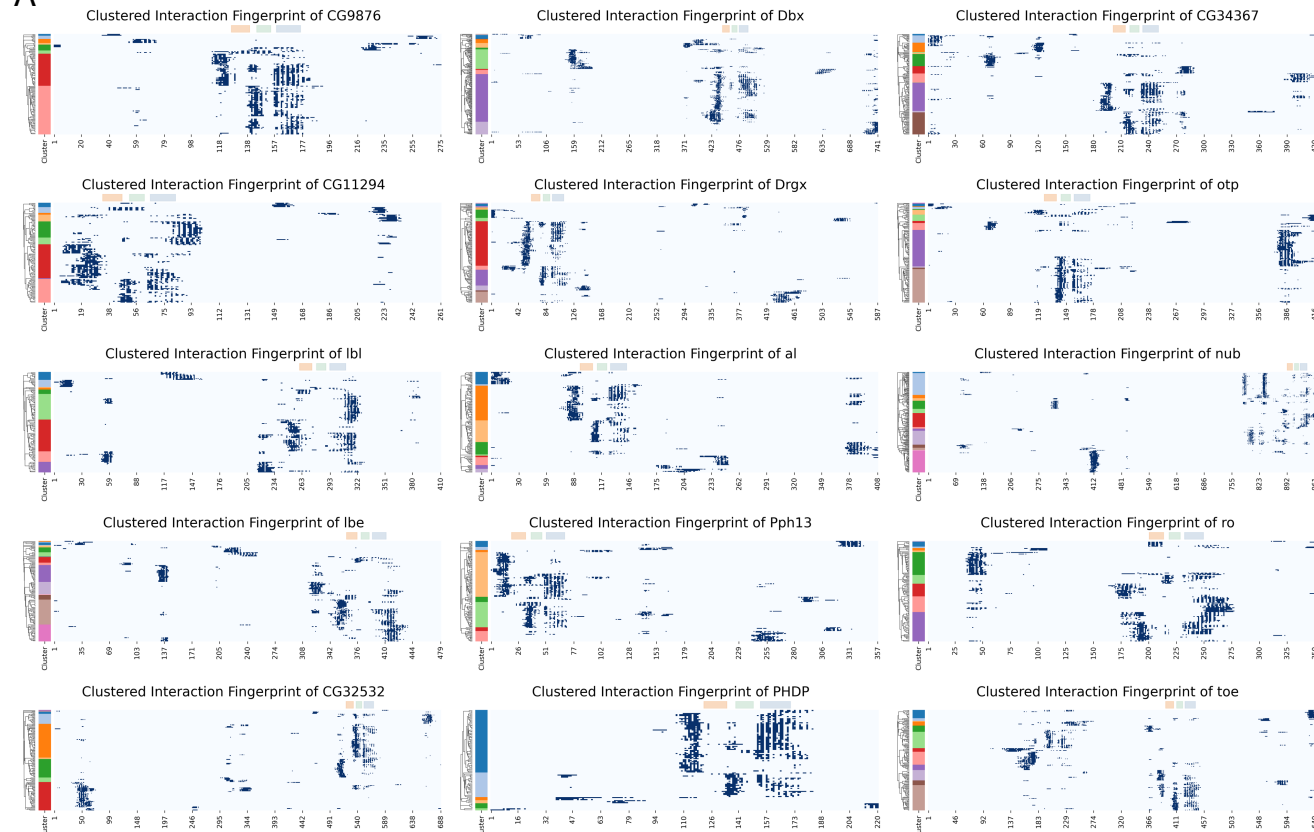

B

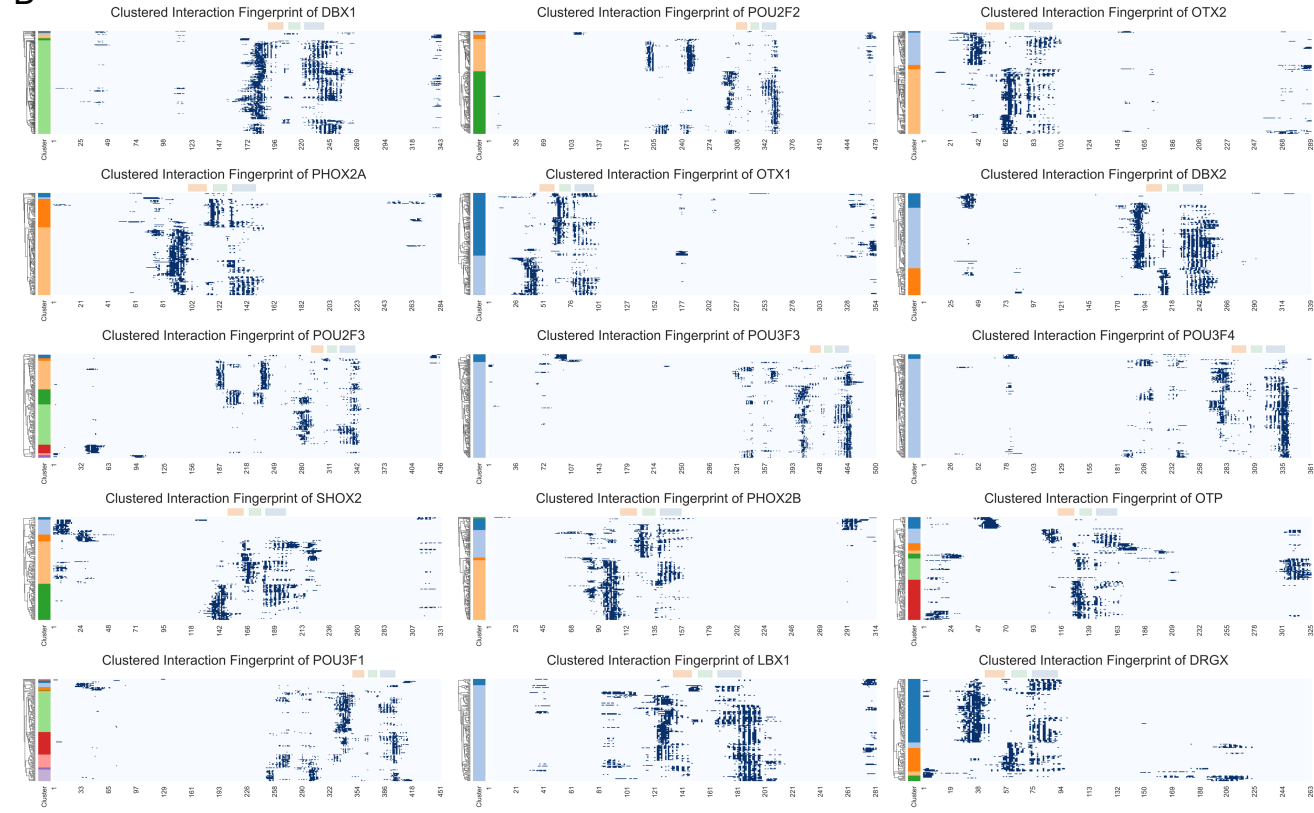

Supplement: Supplement 11 [file media-11.pdf]

**Supplementary Figure 11**

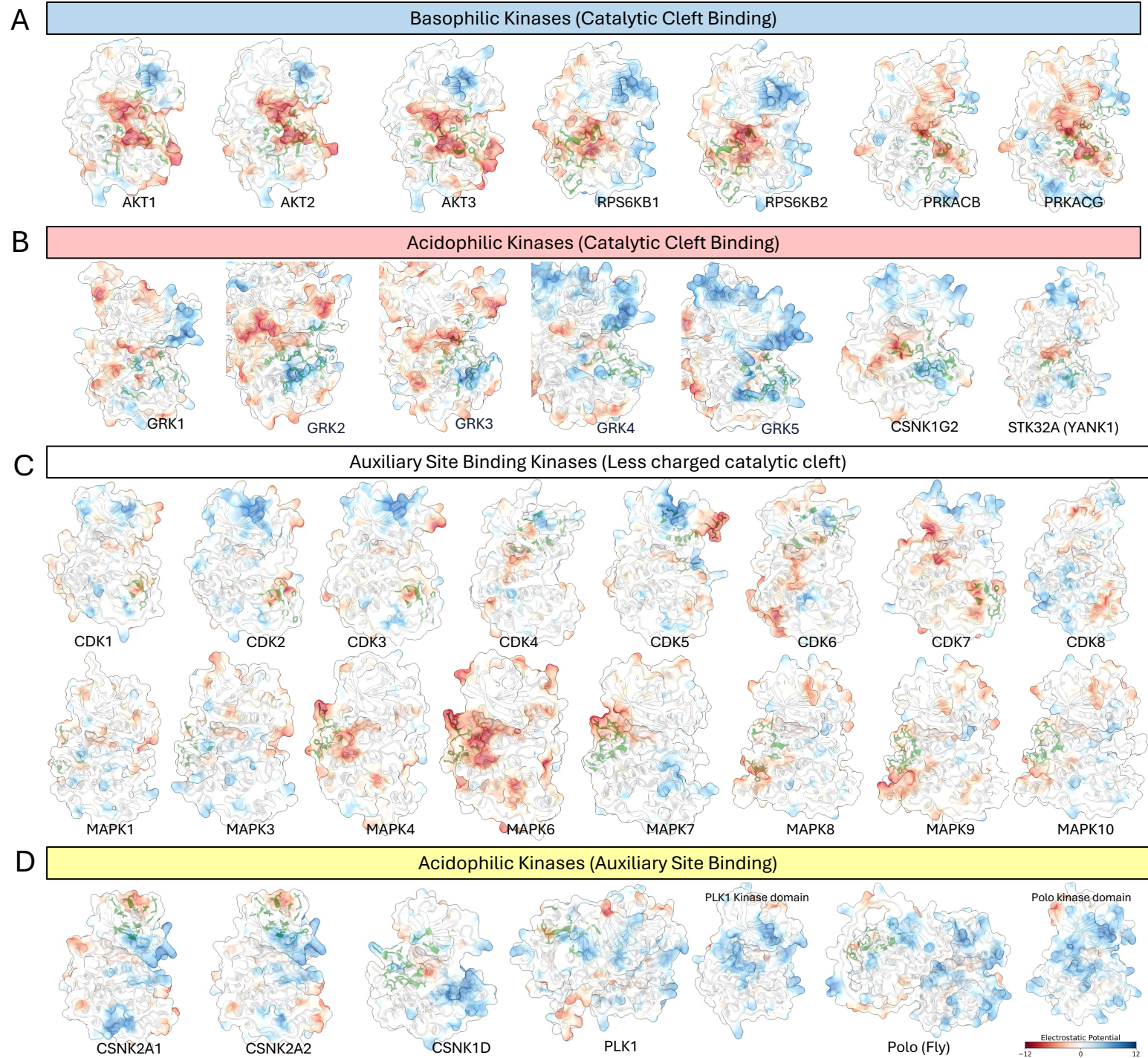

Supplement: Supplement 12 [file media-12.pdf]
