# Supplementary material for: A Structure-Guided Kinase–Transcription Factor Interactome Atlas Reveals Docking Landscapes of the Kinome
Source: bioRxiv. 2025 Nov 25:2025.10.10.681672. Preprint. [Version 4] doi: 10.1101/2025.10.10.681672 (PMC12632555; doi:10.1101/2025.10.10.681672)

Supplementary Figure 2

Literature-based PPIs used as Positive Reference Set in yeast Y2H screen (Yu et al. 2008)

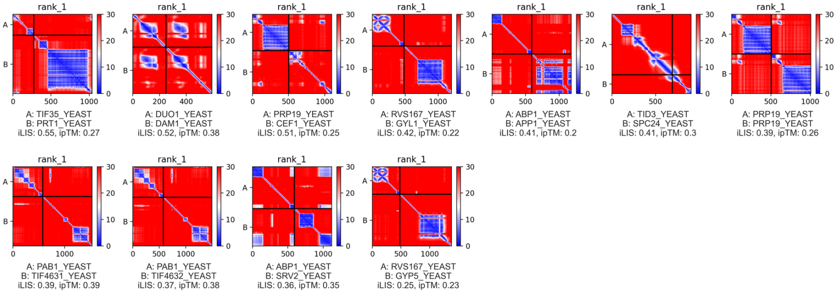

Literature-based PPIs used as Positive Reference Set in fly Y2H screen (Tang et al. 2023)

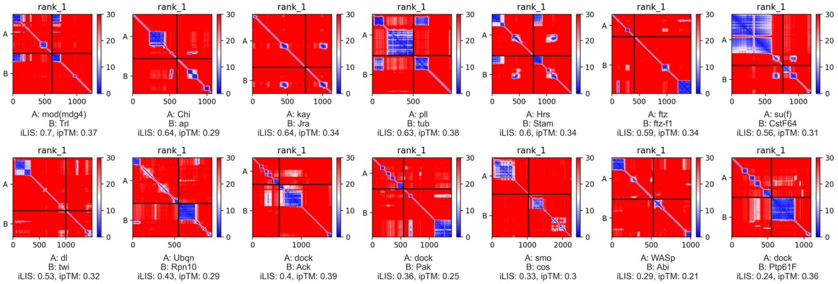

Literature-based PPIs used as Positive Reference Set in human Y2H screen (Braun et al. 2009)

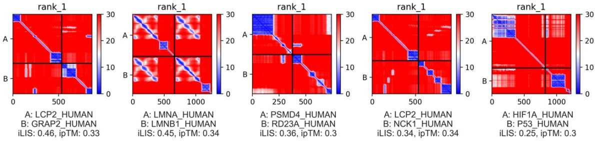

Structure-based PPIs annotated in Eukaryotic Linear Motif database (Kumar et al. 2024)

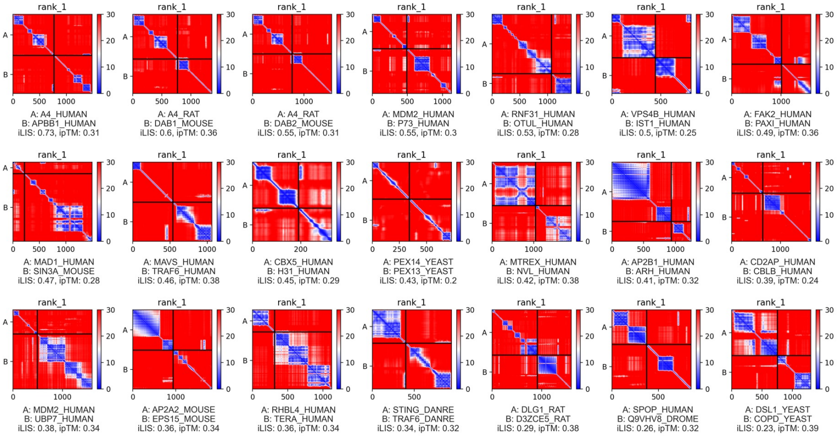

Supplement: Supplement 2 [file media-2.pdf]
