# Supplementary material for: A Structure-Guided Kinase–Transcription Factor Interactome Atlas Reveals Docking Landscapes of the Kinome
Source: bioRxiv. 2025 Nov 25:2025.10.10.681672. Preprint. [Version 4] doi: 10.1101/2025.10.10.681672 (PMC12632555; doi:10.1101/2025.10.10.681672)

Supplementary Figure 8

Cluster 1 (14 members):
CG6800, Cdk1, Cdk12, Cdk2, Cdk4, Cdk5, Cdk7, Cdk8, Cdk9, Eip63E, Pitslre, Sdr, cdc2rk, msn

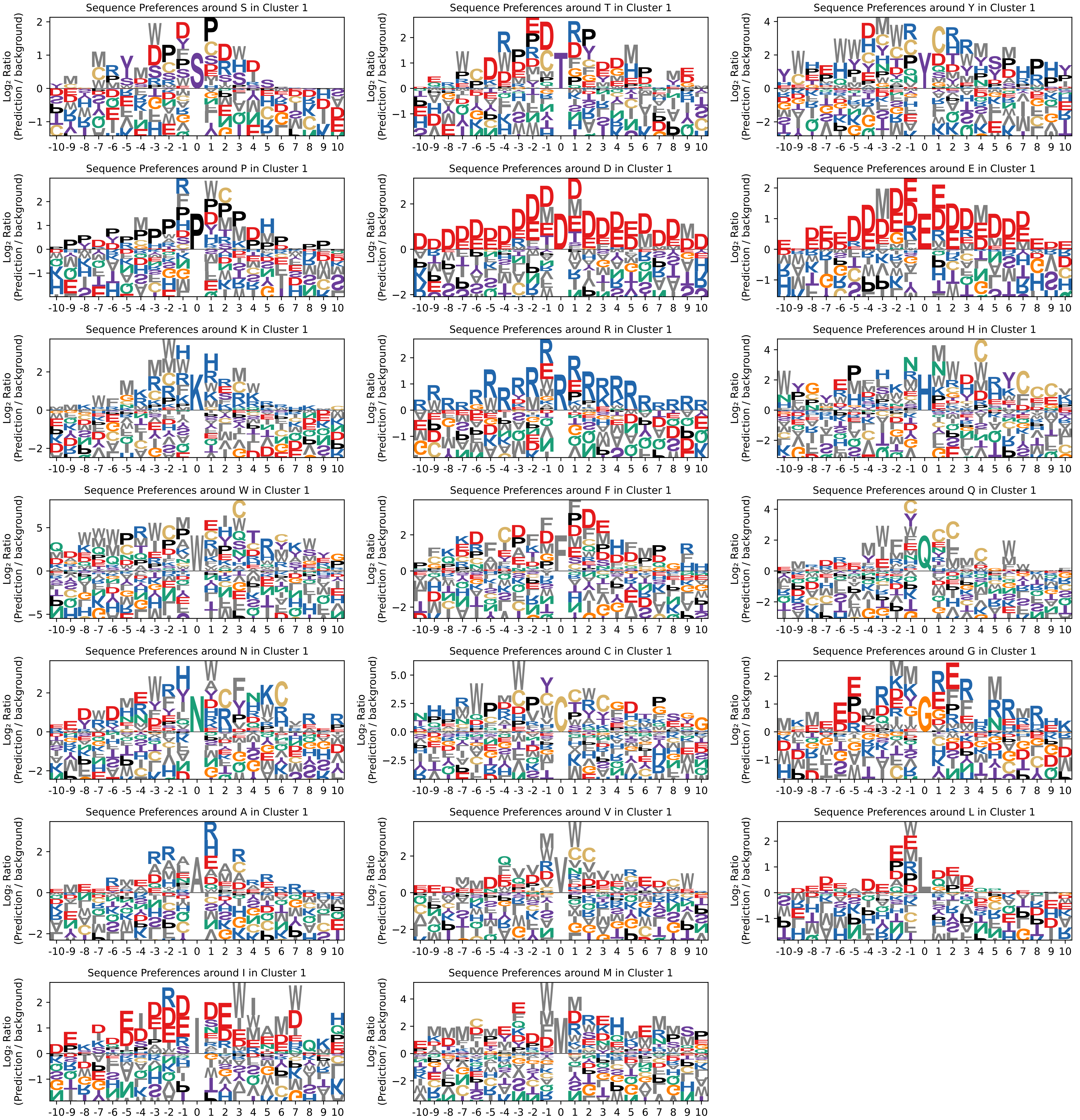

Supplement: Supplement 9 [file media-9.pdf]
