# Supplementary material for: A Structure-Guided Kinase–Transcription Factor Interactome Atlas Reveals Docking Landscapes of the Kinome
Source: bioRxiv. 2025 Nov 25:2025.10.10.681672. Preprint. [Version 4] doi: 10.1101/2025.10.10.681672 (PMC12632555; doi:10.1101/2025.10.10.681672)

Supplementary Figure 12

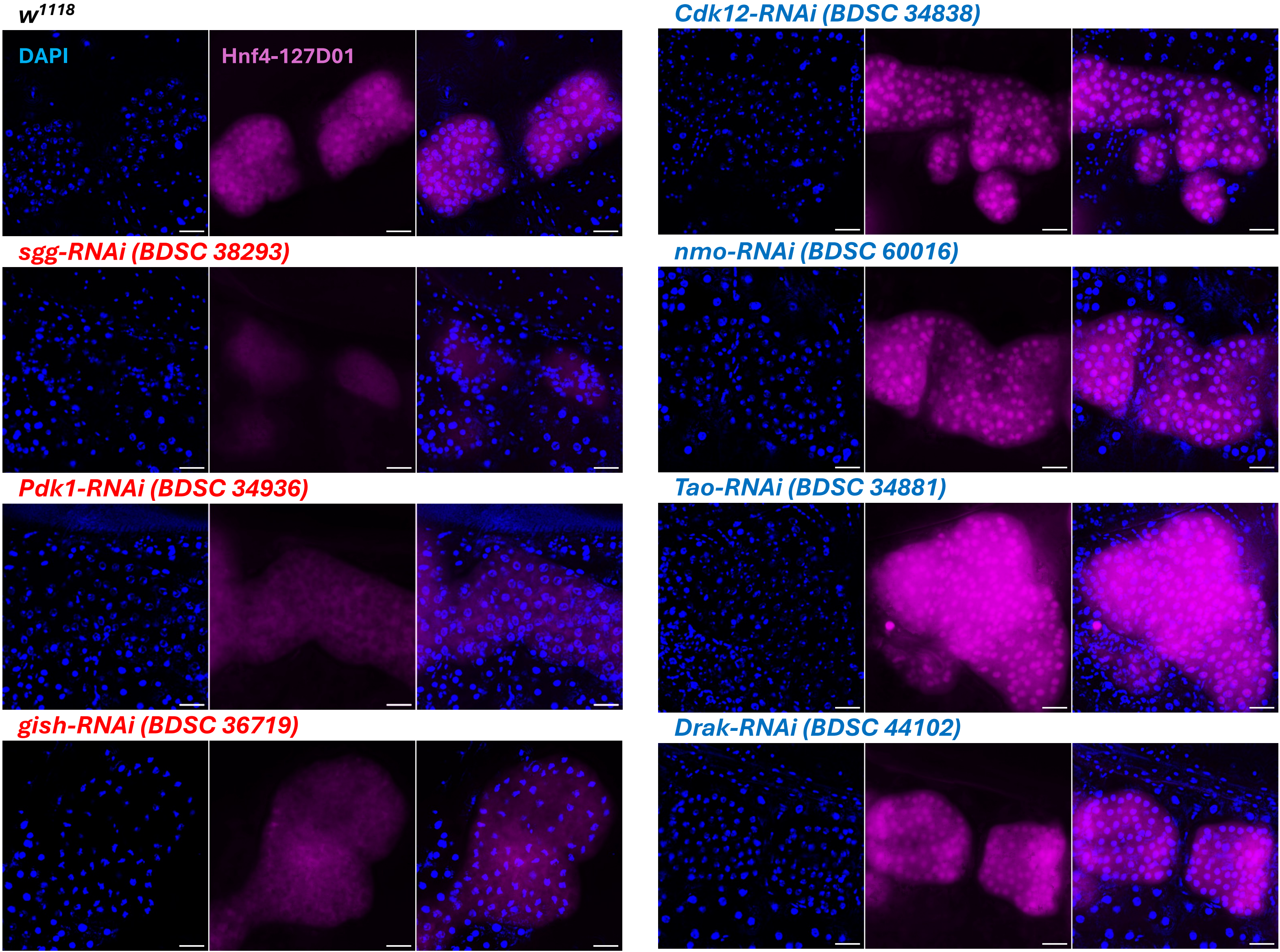

Supplement: Supplement 13 [file media-13.pdf]
